# Bidirectional Mediation and Synergistic Mortality Risks in Diabetes and Cardiovascular Disease: Evidence From NHANES 2005–2018

**DOI:** 10.1155/jdr/8517492

**Published:** 2025-11-10

**Authors:** Zixuan Li, Rong Sun, Tiantian Huang, Zhoubo Han, Xiuping Xuan, Chenghu Huang

**Affiliations:** ^1^Department of Endocrinology, Bishan Hospital of Chongqing Medical University, Bishan Hospital of Chongqing, Chongqing, China; ^2^Department of Ophthalmology, Taihe Hospital, Hubei University of Medical, Shiyan, China; ^3^Department of Endocrinology, The First Affiliated Hospital of Guangxi Medical University, Nanning, China

**Keywords:** bidirectional, cardiovascular disease, causal mediation analysis, diabetes, mortality, synergistic interaction analysis

## Abstract

**Background:**

Diabetes mellitus (DM) and cardiovascular disease (CVD) are interconnected conditions that significantly contribute to global mortality, yet their bidirectional relationship and combined mortality impact remain underexplored.

**Methods:**

Utilizing data from the NHANES 2005–2018 cohort (*N* = 24,934), we categorized participants aged ≥ 35 years into four groups: nondiabetic/non–pre-existing CVD, diabetic/non–pre-existing CVD, nondiabetic/pre-existing CVD, and diabetic/pre-existing CVD. Propensity score matching (PSM) and causal mediation analysis were employed to assess independent and synergistic mortality risks.

**Results:**

Over a mean follow-up of 7.37 years, diabetic/pre-existing CVD participants exhibited the highest mortality rates (61.37 all-cause and 23.88 cardiovascular deaths per 1000 person-years). Diabetes alone increased all-cause mortality by 34% (HR = 1.34, 95% CI = 1.22–1.47) and cardiovascular mortality by 32% (HR = 1.32, 1.10–1.58), while pre-existing CVD alone increased risks by 72% (HR = 1.72, 1.56–1.89) and 142% (HR = 2.42, 2.05–2.87), respectively. Comorbid diabetes/pre-existing CVD synergistically elevated all-cause mortality by 142% (HR = 2.42, 2.19–2.68) and cardiovascular mortality by 237% (HR = 3.37, 2.83–4.02). Although no statistically significant multiplicative interaction was observed, additive interaction metrics between diabetes and pre-existing CVD on mortality risks revealed a stronger synergistic effect on cardiovascular mortality (RERI = 0.64–1.17, AP = 12.01%–23.82%) than on all-cause mortality (RERI = 0.39–0.75, AP = 9.26%–18.73%). Mediation analysis demonstrated bidirectional effects: Diabetes mediated 6.82% of all-cause and 4.17% of cardiovascular mortality in pre-existing CVD patients, while pre-existing CVD mediated 5.47% and 7.87% in diabetic individuals.

**Conclusions:**

Diabetes and pre-existing CVD independently and synergistically increase mortality risks, with additive interactions particularly pronounced for cardiovascular mortality. The bidirectional mediation effects highlight the need for integrated management strategies to mitigate the compounded mortality burden.

## 1. Introduction

Diabetes mellitus, a chronic metabolic disorder characterized by hyperglycemia, has become one of the most serious and major public health concerns. According to the diabetes atlas of the International Diabetes Federation (IDF), approximately 537 million adults aged 20–79 years were living with diabetes in 2021, a figure projected to rise to 783 million by 2045 [1]. This epidemic is not merely a standalone condition but a catalyst for systemic vascular damage, with cardiovascular disease (CVD) accounting for over 50% of diabetes-related mortality [1]. Individuals with Type 2 diabetes (T2D) face a 2–4-fold increased risk of coronary artery disease, stroke, and heart failure compared to nondiabetic counterparts [2, 3]. The multinational CAPTURE study revealed that 34.8% of patients attending routine healthcare visits had established CVD, with the majority (85.8%) classified as atherosclerotic CVD (ASCVD) [4]. Conversely, CVD itself predisposes individuals to dysglycemia. Heart failure population exemplifies this synergy, with 50% prevalence of diabetes and 18% prediabetes [5]. The interplay between diabetes and CVD represents a vicious cycle: Hyperglycemia accelerates atherosclerosis through mechanisms like oxidative stress and chronic inflammation, while pre-existing CVD exacerbates insulin resistance and metabolic dysregulation [6, 7]. Despite advances in pharmacotherapy, the dual burden of these conditions continues to drive premature mortality, reducing life expectancy by 10–20 years in diabetic populations [8].

The mortality ramifications of this interplay are profound. Cardiovascular events mediate 44% of deaths in Type 1 diabetes and 52% in T2D [9]. Diabetes elevates the risk of all-cause mortality by 1.3- to 3.2-fold and cardiovascular mortality by 1.5- to 1.7-fold [10–12]. Beyond cardiovascular outcomes, diabetes-related all-cause mortality also results from cancer, respiratory disease, liver disease, and diabetic ketoacidosis or coma [12]. Notably, even prediabetes, often dismissed as a “borderline” condition, contributes to measurable mortality: increased risk of all-cause (HR = 1.08) and cardiovascular mortality (RR = 1.10) [12]. Despite robust mortality associations, diabetes and CVD are often studied as independent risk factors, neglecting their synergistic mediation effects. For instance, while the UK Prospective Diabetes Study (UKPDS) demonstrated that a 1% reduction in hemoglobin A1c (HbA1c) lowers myocardial infarction risk by 14% [13], it did not evaluate how pre-existing CVD modulates this glycemic benefit. Furthermore, reliance on clinical trial cohorts—often limited by stringent eligibility criteria—restricts the generalizability of such findings to broader populations [14, 15]. Critically, the mechanistic pathways underlying this bidirectional relationship, particularly their mediation effects on population-level mortality, remain inadequately characterized.

In this retrospective cohort-based exploratory analysis utilizing NHANES data, we examined the clinical characteristics of patients with diabetes and pre-existing CVD. We further investigated the mediated effects of pre-existing CVD on diabetes-related all-cause and cardiovascular mortality, as well as explored the role of diabetes in influencing mortality outcomes among individuals with pre-existing CVD. As the first study to apply propensity score matching (PSM) and causal mediation analysis in this context, our research provides a comprehensive evaluation of the bidirectional mechanisms underlying the relationship between diabetes and cardiovascular mortality.

## 2. Materials and Methods

### 2.1. Study Design and Population in NHANES

Data in this retrospective cohort study was the continuous NHANES database from 2005 to 2018, a survey conducted by the National Center for Health Statistics of the Centers for Disease Control and Prevention (CDC) (https://wwwn.cdc.gov/nchs/nhanes/). NHANES data were collected from a nationally representative sample of the civilian noninstitutionalized US population using a multistage probability design.

Questionnaire, laboratory testing, and physical examination data are available. Considering the impact of age on the incidence of CVDs, individuals aged ≥ 35 years old received the assessment of diabetes, and pre-existing CVDs were initially included [16]. Since NHANES was approved by the Institutional Review Board (IRB) of the NCHS of the US CDC and the data were publicly available, no ethical approval of our IRB was required.

### 2.2. Assessment of Diabetes and Pre-Existing CVD Status in NHANES

The self-reported NHANES multiple choice question (MCQ) was considered to have a pre-existing CVD: “Have you ever been told you had (congestive) heart failure, coronary heart disease, angina/angina pectoris, heart attack, or stroke.”

Diabetes status was classified as “non-diabetes” or “diabetes,” a definition that included both diagnosed and undiagnosed conditions. Diagnosed diabetes was determined by a self-reported “yes” to the NHANES question: “Has a doctor ever told you that you have diabetes?” Participants without such a diagnosis were classified as having diabetes if they had a fasting plasma glucose (FPG) ≥ 7.0 mmol/L and an HbA1c ≥ 6.5% or were using hypoglycemic drugs.

### 2.3. Assessment of Mortality Status in NHANES

The study outcome was all-cause mortality and cardiovascular mortality. Mortality status was determined through linkage to the National Death Index from baseline (1999–2004) until December 31, 2019. The follow-up ended with patients' death during 2005–2018 or on December 31, 2019. Cardiovascular mortality was defined as heart disease or cerebrovascular disease listed as the underlying cause of death in death certificates. The National Center for Health Statistics has carefully merged this database with the National Death Index using a detailed probabilistic matching process. We used the International Statistical Classification of Diseases, 10th Revision (ICD-10) to pinpoint deaths from specific causes.

### 2.4. Potential Confounders in NHANES

We merged the continuous NHANES 2005–2018 to ensure a large and representative sample. Sex was classified as “man” or “woman”; race/ethnicity was categorized as “Mexican American,” “non-Hispanic White,” “non-Hispanic Black,” or “other race/ethnicity”; education level was classified as “less than high school,” “high school diploma,” or “more than high school”; marital status was categorized as “married or living with partner” or “single.” Of health-related variables, smoking was categorized as follows: Individuals with a lifetime smoking history of at least five packs of cigarettes (equivalent to 100 cigarettes) or more were classified as “smokers,” and those with a smoking experience of less than five packs of cigarettes and were currently nonsmoking were designated “nonsmokers.” Alcohol drinking was binary with individuals consuming alcohol once a month or more for the past year classified as “drinkers” and others as “nondrinkers.” Physical activity was categorized by metabolic equivalent hours per week (MET min/week) of moderate-to-vigorous physical activity and was defined as “less active (< 600 MET min/week)” or “active (≥ 600 MET min/week)” [17]. The self-reported NHANES blood pressure and cholesterol (BPQ) was used to assess hypertension: “Ever told you had high blood pressure.” Individuals who have a positive answer to the BPQ or taking hypertension drugs were also recognized as patients with hypertension. Dyslipidemia was also assessed by BPQ: “Doctor told you - high cholesterol level.” Individuals who have a positive answer to the BPQ or taking dyslipidemia drugs were also recognized as patients with dyslipidemia. We calculated body mass index (BMI) as measured weight (in kilograms) divided by height (in meters squared) and categorized participants as normal weight (BMI < 25 kg/m^2^), overweight (BMI 25–29.9 kg/m^2^), and obese (BMI ≥ 30 kg/m^2^). Income was measured based on the poverty income ratio, an index reflecting the ratio of household income to the household poverty level determined by area of residence and household size. Low family income was defined as a ratio below 1. We excluded the missing confounders including education level, marital status, smoking, hypertension, dyslipidemia, BMI, and low family income. For other missing each continuous covariates (including drinking and physical activity), since it was acceptable to continue our analysis without further evaluation or adjustment if 10% or less of the data are missing, we did not make any changes.

### 2.5. Statistical Analysis

Categorical variables were expressed as percentages. Continuous variables were expressed as means ± standard deviation. The Student *t*-test (for variables normally distributed), the Mann–Whitney *U* test (for variables nonnormally distributed), and the chi-square test (for categorical variables) were used to compare the differences among groups according to diabetes and pre-existing CVD status. Baseline characteristics were cross-tabulated by each group.

To address potential imbalances in patient characteristics, we performed PSM using nondiabetic participants without pre-existing CVD as the anchor control group. A caliper width equal to 0.01 standard deviation of the logit of the propensity score was applied to perform 1:1 matching between the diabetic group without pre-existing CVD and the anchor control group. The propensity model incorporated the following confounding variables: sex, age, race/ethnicity, education level, marital status, smoking status, low family income, BMI, hypertension, and dyslipidemia. These variables were selected based on their established relevance to all-cause and cardiovascular mortality [6, 18]. Drinking status and physical activity were excluded due to a substantial proportion of missing data. Matched controls were removed from the pool to ensure matching without replacement. This process was repeated iteratively for the remaining groups (e.g., nondiabetic participants with pre-existing CVD and diabetic participants with pre-existing CVD) against the remaining anchor subjects. All successfully matched participants were then combined to form a balanced postmatching cohort (post-PSM cohort). Covariate balance between groups pre- and pro-PSM was evaluated using standardized differences, with a threshold greater than 0.01 indicating meaningful imbalance.

We assessed the proportional hazards assumption by testing the significance of the interaction term between diabetes status and the history of pre-existing CVD and confirmed that it was not violated. We also calculated relative excess risk due to interaction (RERI), attributable proportion (AP), and synergy index (SI), as previously documented [19, 20]. We conducted several sensitivity analyses to test the robustness of our primary findings.

When performing NHANES analysis, we implemented multivariate-adjusted logistic regression to assess the association of diabetes and pre-existing CVD with all-cause and cardiovascular mortality. Four models adjusted for covariates were assessed: Crude model was not adjusted; in Model 1, we adjusted sex, age, and race/ethnicity; Model 2 was adjusted for the covariates in Model 1 as well as education level, mortal statue, low family income, BMI, hypertension, dyslipidemia, and smoking status; Model 3 was adjusted for the covariates in Model 2 plus drinking status and physical activity. Results are presented as odds ratios (ORs) or *β* coefficients (95% confidence interval [CI]). Given the complex probabilistic clustering design of NHANES, weights were considered in statistical analyses in this study. R software Version 4.1.3 (R Foundation, Vienna, Austria) and EmpowerStats software (X&Y Solutions Inc., Boston, Massachusetts, United States) were used to perform all statistical analyses.

## 3. Results

### 3.1. Main Characteristics of Participants

**Figure 1:**
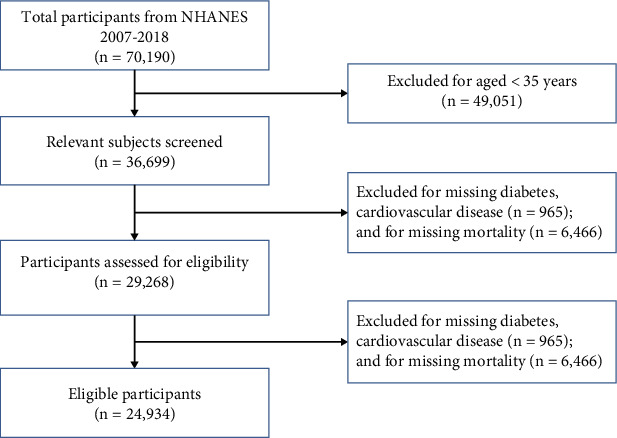
Flow chart showing the selection of the study sample from the continuous National Health and Nutrition Examination Survey (NHANES).

Figure 1 depicts the flowchart for participant screening. A total of 24,934 individuals aged ≥ 35 were included (Figure 1). The survival group had a mean age of 54.89 ± 12.74 years, while the all-cause mortality group averaged 70.34 ± 11.28 years (Table 1). Among the survival group, 32.85% had diabetes, and 10.80% had pre-existing CVD. In contrast, 38.91% of the all-cause mortality group had diabetes, and 36.02% had pre-existing CVD (Table 1). Among cardiovascular mortality cases, 46.26% had diabetes, and 40.85% had pre-existing CVD (Table S1).

Participants with both diabetes and pre-existing CVD were the oldest (67.37 ± 10.71 years) and had the highest BMI (32.19 ± 7.74 kg/m^2^) (Table 2). Significant differences were observed in covariates such as sex, race/ethnicity, education level, marital status, smoking, low family income, physical activity, hypertension, and dyslipidemia among the four groups (nondiabetic participants without pre-existing CVD, diabetic without pre-existing CVD, nondiabetic with pre-existing CVD, and diabetic with pre-existing CVD) (Table 2). According to the status of diabetes or the presence of pre-existing evidence of CVD at baseline, the differences of these covariates were also significant (Tables S2 and S3).

We identified three pairwise 1:1 propensity score matched cohorts of nondiabetic participants without pre-existing CVD or diabetes without pre-existing (*n* = 6406 pairs), or nondiabetes with pre-existing CVD (*n* = 1715 pairs), or diabetes with pre-existing CVD (*n* = 1736 pairs) (Table S4 and Figure 1). Overall, there were 17,320 unique participants contributing to the three pairs matched on propensity score (Table S4).

After PSM, the mean follow-up time was 6.93 ± 3.84 years for all groups and persistent statistically significant disparities in both all-cause and cardiovascular mortality across the four subgroups (Table 3 and Tables S5–S8). And compared with other groups, diabetic participants with pre-existing CVD were generally older, lower education level, higher proportion of hypertension, dyslipidemia, and obesity (BMI ≥ 30 kg/m^2^) (Table 3).

### 3.2. Respective Predictive Value of Diabetes and Pre-Existing CVD

The mean follow-up duration was 7.37 ± 3.93 years. The overall all-cause and cardiovascular mortality rates were 18.85 and 5.74 per 1000 person-years, respectively. The incidence of all-cause mortality increased progressively across groups: 12.22 (nondiabetic without pre-existing CVD), 18.35 (diabetic without pre-existing CVD), 51.75 (nondiabetic with pre-existing CVD), and 61.37 per 1000 person-years (diabetic with pre-existing CVD). Similarly, cardiovascular mortality rates were 3.10‰, 4.78‰, 20.37‰, and 23.88‰ per 1000 person-years, respectively (Table 4). After full adjustment, diabetes was associated with a 1.34- to 1.38-fold increased risk of all-cause mortality and a 1.29- to 1.41-fold increased risk of cardiovascular mortality, regardless of pre-existing CVD status. Pre-existing CVD significantly increased the risk of all-cause mortality (HRs: 1.65–1.92) and cardiovascular mortality (HRs: 2.25–2.87), depending on diabetic status (Table 4).

**Figure 2:**
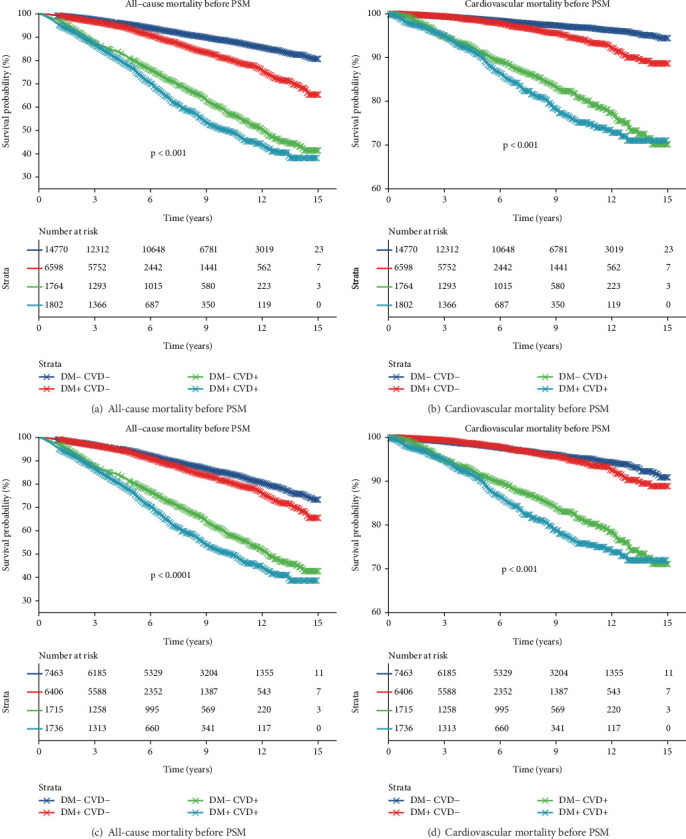
Kaplan–Meier survival curves according to the status of diabetes and pre-existing CVD on all-cause and cardiovascular mortality. CVD, cardiovascular disease; PSM, propensity score matching.

The Kaplan–Meier analysis showed significant differences in survival time among the four groups, with the shortest survival time observed in diabetic participants with pre-existing CVD, followed by nondiabetic participants with pre-existing CVD, diabetic participants without pre-existing CVD, and nondiabetic participants without pre-existing CVD (Figure 2). These findings were consistent in pre- and post-PSM analysis (Figure 2).

### 3.3. Joint Influences of Diabetes and Pre-Existing CVD on the Mortality Risks

Using nondiabetic participants without pre-existing CVD as a reference, the risk of all-cause mortality increased by 34% in diabetic participants without pre-existing CVD (HR 1.34, 95% CI: 1.22–1.47), by 72% in nondiabetic participants with pre-existing CVD (HR 1.72, 95% CI: 1.56–1.89), and by 142% in diabetic participants with pre-existing CVD (HR 2.42, 95% CI: 2.19–2.68). For cardiovascular mortality, the results were sustained as being statistically significant in the different groups after all adjustment (in diabetic participants without pre-existing CVD HR 1.32, 95% CI: 1.10–1.58; non-diabetic participants with pre-existing CVD HR 2.42, 95% CI: 2.05–2.87; diabetic participants with pre-existing CVD HR 3.37, 95% CI: 2.83–4.02) (Table 5). These results might demonstrate that diabetes and pre-existing CVD mutually increased the risks of all-cause and cardiovascular mortality.

Post-PSM analysis confirmed increased effects of diabetes and pre-existing CVD on all-cause and cardiovascular mortality. The Cox proportional hazards regression analysis still indicated a significant uptrend in all-cause mortality risk for diabetic participants without pre-existing CVD HR 1.35 (95% CI: 1.22–1.49), nondiabetic participants with pre-existing CVD HR 1.74 (95% CI: 1.57–1.93), and diabetic participants with pre-existing CVD HR 2.55 (95% CI: 2.29–2.83) (Table 6). For the cardiovascular mortality risk, in full adjusted, an increase was with a 0.22-fold in diabetic participants without pre-existing CVD (HR 1.22, 95% CI: 1.01–1.48), 1.31-fold in nondiabetic participants with pre-existing CVD (HR 2.31, 95% CI: 1.94–2.73), and 2.34-fold in diabetic participants with pre-existing CVD (HR 3.34, 95% CI: 2.78–4.00) (Table 6). These findings emphasized the compounded mortality burden of comorbid diabetes and pre-existing CVD.

### 3.4. Subgroup Analysis

Generally, participants who had both diabetes and pre-existing CVD independently elevated risks of all-cause and cardiovascular mortality, demonstrating significant combined influences (Tables S9 and S10). Subgroup analysis showed that diabetic participants with pre-existing CVD were more significantly associated with adverse outcomes, particularly in younger (< 65 years: HR = 7.08 all-cause mortality, 12.94 cardiovascular mortality) and nonhypertensive participants (HR = 9.94 and 20.81, respectively) (Tables S9 and S10).

### 3.5. Interaction Between Diabetes and Pre-Existing on Mortality Risk

#### 3.5.1. Synergistic Effects of Diabetes and Pre-Existing CVD

Despite significant interaction between diabetes and pre-existing CVD both on the risk of all-cause and cardiovascular mortality in the crude model, the interaction disappeared in full adjustment and post-PSM analysis (Tables 5 and 6). In pre-PSM and post-PSM analysis, there was a strong additive interaction between diabetes and pre-existing CVD both on the risk of all-cause (RERI range: 0.39–0.75) and cardiovascular mortality (RERI range: 0.64–1.17). The AP derived from the relationship in the risk of all-cause mortality (AP range: 9.26%–18.73%) was significantly lower than that in the risk of cardiovascular mortality (AP range: 12.01%–23.82%) (Tables 5 and 6). And SI confirmed these synergistic effects of diabetes and pre-existing CVD (Tables 5 and 6). These results suggested that their synergistic effects might be additive rather than multiplicative.

#### 3.5.2. Mediated Effects of Diabetes and Pre-Existing CVD

##### 3.5.2.1. Diabetes as Mediator Between Pre-Existing CVD and Mortality

**Figure 3:**
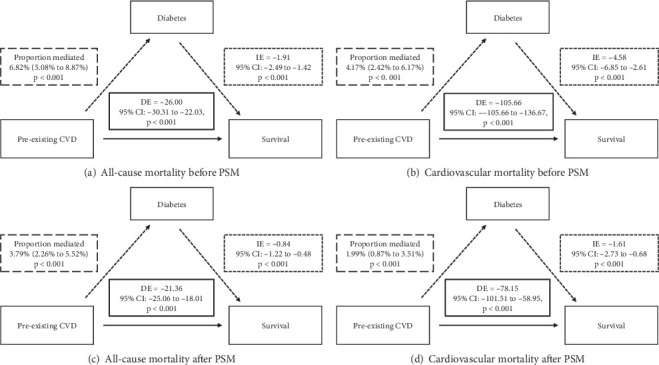
Diabetes as mediator between pre-existing CVD and mortality. The solid line represented the direct effect (DE), and the dotted line represented the indirect effect (IE). CVD, cardiovascular disease; PSM, propensity score matching.

We further investigated the interplay between diabetes and pre-existing CVD by causal mediation analysis. We firstly found that diabetes significantly mediated all-cause mortality both before (proportion mediated: 6.82%, 95% CI: 5.08%–8.87%) and after PSM (3.79%, 95% CI: 2.26%–5.52%). For cardiovascular mortality, mediation was significant both pre-PSM (4.17%, 95% CI: 2.42%–6.17%) and post-PSM (1.99%, 95% CI: 0.87%–3.51%) (Figure 3). These findings suggested that diabetes mediated the relationship between pre-existing CVD and all-cause mortality and cardiovascular mortality.

##### 3.5.2.2. Pre-Existing CVD as Mediator Between Diabetes and Mortality

**Figure 4:**
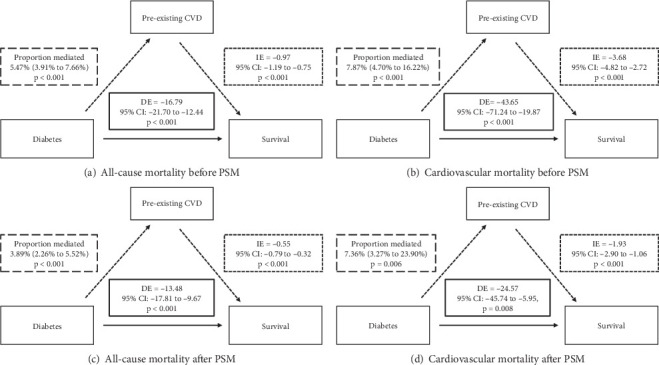
Pre-existing CVD as mediator between diabetes and mortality. The solid line represented the direct effect (DE), and the dotted line represented the indirect effect (IE). CVD, cardiovascular disease; PSM, propensity score matching.

Pre-existing CVD significantly mediated diabetes-associated all-cause mortality risk pre-PSM (5.47%, 95% CI: 3.91%–7.66%) and post-PSM (3.89%, 95% CI: 2.26%–5.52%). For cardiovascular mortality, mediation was significant both pre-PSM (7.87%, 95% CI: 4.70%–16.22%) and post-PSM (7.35%, 95% CI: 3.27%–23.90%) (Figure 4). These results indicated that pre-existing CVD mediates both all-cause and cardiovascular mortality risks associated with diabetes.

## 4. Discussion

This study provided a comprehensive analysis of the bidirectional relationship between diabetes and pre-existing CVD and their combined impact on all-cause and cardiovascular mortality using data from the NHANES 2005–2018 cohort. Our findings highlighted the significant interplay between diabetes and pre-existing CVD, demonstrating that both conditions independently and synergistically increased mortality risks. Mediation analysis further elucidated complex pathophysiological interactions between these comorbidities in mortality pathways. These findings underscored the complex pathophysiological interdependence between metabolic and cardiovascular systems in determining clinical outcomes.

Consistent with previous data [2, 3, 21–23], our results confirmed that both diabetes and pre-existing CVD were strong independent predictors of all-cause and cardiovascular mortality. Diabetes alone conferred a 1.31–1.33-fold increased risk of all-cause mortality and 1.29–1.30-fold risk of cardiovascular mortality (Table 4). Comparatively, pre-existing CVD showed more pronounced associations, with 1.62–1.80-fold and 2.35–2.69-fold elevated risks for all-cause and cardiovascular mortality, respectively. Notably, coexisting conditions potentiated mortality risks, reaching HR of 5.08 for all-cause and 4.37 for cardiovascular mortality (Tables 5 and 6), indicating a complex relationship between diabetes and pre-existing CVD in mortality risks beyond the simple sum of risks [22, 24].

We further explored the mutual effects between diabetes and pre-existing CVD related to the mortality risks by comprehensive interaction and mediation analyses. We firstly reported that diabetes mediated 6.82% of all-cause and 4.17% of cardiovascular mortality in participants with pre-existing CVD (Figure 3), while pre-existing CVD mediated 5.47% and 7.87%, respectively, in diabetics (Figure 4). Crucially, additive interaction metrics—including RERI (range: 0.39–1.17), AP (9.26%–23.82%), and SI (1.12–1.54)—demonstrated that the synergistic effects between diabetes and pre-existing CVD on mortality risks were additive rather than multiplicative (Tables 5 and 6). Pathophysiological context suggests that this reciprocity originates from shared mechanisms: Diabetic hyperglycemia promotes endothelial dysfunction via advanced glycation end products (AGEs) and reactive oxygen species (ROS), while CVD-induced hypoxia and neurohormonal activation impair pancreatic *β*-cell function [25–27]. This vicious cycle explains the observed bidirectional mediation, particularly evident in heart failure populations where 50% prevalence of diabetes coexists with 18% prediabetes [5].

The findings of this study had several important implications for clinical practice. Clinicians should recognize that diabetes and pre-existing CVD are not isolated comorbidities but interconnected drivers of mortality. Routine screening for CVD in diabetic patients—and vice versa—could enable earlier interventions [28]. Tools like coronary artery calcium scoring or assessment of subclinical atherosclerosis may identify subclinical CVD in asymptomatic diabetics, while HbA1c monitoring in CVD patients could detect dysglycemia before overt diabetes develops [13, 29]. Besides, the bidirectional mediation effects emphasize the need for holistic care models targeting both diabetes and CVD [30]. For example, sodium-glucose cotransporter-2 inhibitor (SGLT2i) and glucagon-like peptide-1 receptor agonists (GLP-1 RAs), which improve glycemic control while reducing cardiovascular events, should be prioritized in high-risk populations [21, 31]. Similarly, cardiovascular rehabilitation programs should incorporate glycemic monitoring to break the cycle of mutual risk amplification [32]. In addition, the synergistic effects of diabetes and pre-existing CVD on mortality highlight the need for personalized risk assessment tools that account for the presence of both conditions [30]. Clinicians should consider the combined impact of diabetes and CVD when stratifying patients' mortality risks and tailoring treatment plans.

Moreover, subgroup analyses indicated that these synergistic effects were especially marked among younger adults (aged < 65 years) and nonhypertensive individuals. Consistently, a territory-wide diabetes complication screening program in Hong Kong, which included 360,202 Chinese patients with T2D, revealed that although older adults had a higher absolute mortality risk, the relative risk of all-cause mortality associated with most risk factors was greater in younger patients [33]. Younger individuals with T2D are also more likely to exhibit a more adverse cardiometabolic risk profile, characterized by poorer glycemic control, higher rates of overweight/obesity, and dyslipidemia [34–36]. It is well documented that the coexistence of hypertension and T2D is particularly detrimental due to their strong associations with CVD, stroke, renal disease progression, and diabetic nephropathy [33, 34, 37]. These competing risk factors may attenuate the specific synergistic effect of diabetes and pre-existing CVD on mortality.

This study had several strengths, including the use of a large, nationally representative sample from the NHANES database, which enhanced the generalizability of our findings. The application of PSM analysis and mediation analysis allowed us to control for confounding factors and explore the bidirectional relationships between diabetes, pre-existing CVD, and mortality. However, several limitations should be acknowledged. First, the observational nature of the study precluded the establishment of causal relationships. Second, despite the use of PSM, residual confounding might persist due to unmeasured variables, such as the duration of diabetes. It is well-established that longer diabetes duration is associated with an increased risk of CVD and mortality [38]. Third, critical variables including medication use (e.g., SGLT2i and ACE inhibitors), diabetes duration, and specific inflammatory biomarkers (e.g., hs-CRP) were not fully accounted for. SGLT2i and GLP-1 receptor agonists are currently first-line recommended therapies for patients with diabetes and CVD due to their demonstrated benefits in glycemic control and mortality reduction [39]. Similarly, renin-angiotensin system blockers (ACE inhibitors or ARBs), as first-choice treatments for diabetes with hypertension, have been shown to significantly improve prognosis [40]. Additionally, inflammation plays an important role in the pathogenesis and progression of both diabetes and CVD [41]. The absence of these variables may affect both the exposure-outcome relationship and the proposed mediation pathways. Fourth, the reliance on self-reported data for diabetes and pre-existing CVD status might introduce misclassification bias. Finally, the mediation analysis assumed no unmeasured confounding between the mediator and outcome, which may not hold true in all cases.

## 5. Conclusions

In conclusion, this study demonstrated that diabetes and pre-existing CVD were independently and synergistically associated with increased risks of all-cause and cardiovascular mortality. The bidirectional mediation effects also suggested that these conditions formed a lethal synergy requiring integrated care models to disrupt their mutually reinforcing mortality pathways. These findings underscored the importance of integrated management strategies for diabetes and CVD to reduce mortality risks and improve long-term outcomes. Future research should focus on elucidating the underlying mechanisms of these relationships and developing targeted interventions to mitigate the combined burden of diabetes and CVD.

## Figures and Tables

**Table 1:** Characteristics of adults according to all-cause mortality before PSM.

	**Total**	**Survival**	**Morality**	**p** **value**⁣^∗^
*N*	24,934	21,472	3462	
Participants, *N* (%)				< 0.001
Diabetes^−^, pre-existing CVD ^−^	14,770 (59.24%)	13,280 (61.85%)	1490 (43.04%)	
Diabetes^+^, pre-existing CVD ^−^	6598 (26.46%)	5873 (27.35%)	725 (20.94%)	
Diabetes^−^, pre-existing CVD ^+^	1764 (7.07%)	1139 (5.30%)	625 (18.05%)	
Diabetes^+^, pre-existing CVD ^+^	1802 (7.23%)	1180 (5.50%)	622 (17.97%)	
Follow-up (years)	7.37 ± 3.93	7.66 ± 3.92	5.54 ± 3.44	< 0.001
Pre-existing CVD, *N* (%)				< 0.001
No	21,368 (85.70%)	19,153 (89.20%)	2215 (63.98%)	
Yes	3566 (14.30%)	2319 (10.80%)	1247 (36.02%)	
Diabetes, *N* (%)				< 0.001
No	16,534 (66.31%)	14,419 (67.15%)	2115 (61.09%)	
Yes	8400 (33.69%)	7053 (32.85%)	1347 (38.91%)	
Follow-up (years)	7.37 ± 3.93	7.66 ± 3.92	5.54 ± 3.44	< 0.001
Age (years)	57.03 ± 13.63	54.89 ± 12.74	70.34 ± 11.28	< 0.001
Age categorical (years)				< 0.001
< 65	17,119 (68.66%)	16,170 (75.31%)	949 (27.41%)	
≥ 65	7815 (31.34%)	5302 (24.69%)	2513 (72.59%)	
Sex, *N* (%)				< 0.001
Man	8773 (35.18%)	7190 (33.49%)	1583 (45.73%)	
Woman	16,161 (64.82%)	14,282 (66.51%)	1879 (54.27%)	
Race/ethnicity, *N* (%)				< 0.001
Mexican American	3509 (14.07%)	3231 (15.05%)	278 (8.03%)	
Other Hispanic	2257 (9.05%)	2079 (9.68%)	178 (5.14%)	
Non-Hispanic White	11,184 (44.85%)	9031 (42.06%)	2153 (62.19%)	
Non-Hispanic Black	5335 (21.40%)	4616 (21.50%)	719 (20.77%)	
Other race	2649 (10.62%)	2515 (11.71%)	134 (3.87%)	
Education, *N* (%)				< 0.001
Less than high school	6480 (25.99%)	5232 (24.37%)	1248 (36.05%)	
High school diploma	5681 (22.78%)	4768 (22.21%)	913 (26.37%)	
More than high school	12,773 (51.23%)	11,472 (53.43%)	1301 (37.58%)	
Marital status, *N* (%)				< 0.001
Married or living with a partner	15,730 (63.09%)	14,031 (65.35%)	1699 (49.08%)	
Single	9204 (36.91%)	7441 (34.65%)	1763 (50.92%)	—
Drinking status, *N* (%)				0.006
Nondrinkers	15,129 (60.68%)	13,064 (60.84%)	2065 (59.65%)	
Drinkers	7829 (31.40%)	6721 (31.30%)	1108 (32.00%)	
Missing	1976 (7.92%)	1687 (7.86%)	289 (8.35%)	
Smoking status, *N* (%)				< 0.001
Nonsmokers	11875 (47.63%)	9772 (45.51%)	2103 (60.75%)	
Smokers	13,059 (52.37%)	11,700 (54.49%)	1359 (39.25%)	
Physical activity, *N* (%)				< 0.001
Less active	9176 (36.80%)	7220 (33.63%)	1956 (56.50%)	
Active	8756 (35.12%)	7919 (36.88%)	837 (24.18%)	
Missing	7002 (28.08%)	6333 (29.49%)	669 (19.32%)	
BMI (kg/m^2^)	29.53 ± 6.86	29.66 ± 6.83	28.73 ± 6.96	0.011
BMI categorical (kg/m^2^)				< 0.001
< 25	6397 (25.66%)	5323 (24.79%)	1074 (31.02%)	
≥ 25, < 30	8566 (34.35%)	7404 (34.48%)	1162 (33.56%)	—
≥ 30	9971 (39.99%)	8745 (40.73%)	1226 (35.41%)	
Low family income, *N* (%)				< 0.001
No	20,150 (80.81%)	17,438 (81.21%)	2712 (78.34%)	
Yes	4784 (19.19%)	4034 (18.79%)	750 (21.66%)	
Hypertension, *N* (%)				< 0.001
No	11,784 (47.26%)	10,883 (50.68%)	901 (26.03%)	
Yes	13,150 (52.74%)	10,589 (49.32%)	2561 (73.97%)	
Dyslipidemia, *N* (%)				0.729
No	5430 (21.78%)	4661 (21.71%)	769 (22.21%)	
Yes	19,504 (78.22%)	16,811 (78.29%)	2693 (77.79%)	

*Note:* Diabetes −: nondiabetic participants; diabetes +: diabetic participants; pre-existing CVD −: without pre-existing CVD; pre-existing CVD +: with pre-existing CVD.

Abbreviations: BMI, body mass index; CVD, cardiovascular disease; PSM, propensity score matching.

⁣^∗^For continuous variables, *p* value was by survey-weighted linear regression; for categorical variables, *p* value was by survey-weighted chi-square test.

**Table 2:** Participants with diabetic and pre-existing evidence of CVD at baseline in 24,934 subjects before PSM.

	**No pre-existing CVD**		**Pre-existing CVD**		**p** **value**⁣^∗^
	**Nondiabetic**	**Diabetic**	**Nondiabetic**	**Diabetic**	
*N*	14,770	6598	1764	1802	
All-cause mortality, *N* (%)					< 0.001
No	13,280 (89.91%)	5873 (89.01%)	1139 (64.57%)	1180 (65.48%)	
Yes	1490 (10.09%)	725 (10.99%)	625 (35.43%)	622 (34.52%)	
Cardiovascular mortality, *N* (%)					< 0.001
No	14,392 (97.44%)	6409 (97.14%)	1518 (86.05%)	1560 (86.57%)	
Yes	378 (2.56%)	189 (2.86%)	246 (13.95%)	242 (13.43%)	
Follow-up (years)	8.26 ± 3.91	5.99 ± 3.45	6.85 ± 4.00	5.62 ± 3.51	< 0.001
Age (years)	54.18 ± 13.25	57.96 ± 12.72	66.91 ± 12.56	67.37 ± 10.71	< 0.001
Age categorical (years)					
< 65	11,302 (76.52%)	4457 (67.55%)	684 (38.78%)	676 (37.51%)	< 0.001
≥ 65	3468 (23.48%)	2141 (32.45%)	1080 (61.22%)	1126 (62.49%)	
Sex, *N* (%)					< 0.001
Man	5785 (39.17%)	1477 (22.39%)	858 (48.64%)	653 (36.24%)	
Woman	8985 (60.83%)	5121 (77.61%)	906 (51.36%)	1149 (63.76%)	
Race/ethnicity, *N* (%)					< 0.001
Mexican American	1988 (13.46%)	1195 (18.11%)	123 (6.97%)	203 (11.27%)	
Other Hispanic	1253 (8.48%)	754 (11.43%)	93 (5.27%)	157 (8.71%)	
Non-Hispanic White	6996 (47.37%)	2251 (34.12%)	1081 (61.28%)	856 (47.50%)	
Non-Hispanic Black	2971 (20.12%)	1547 (23.45%)	363 (20.58%)	454 (25.19%)	
Other race	1562 (10.58%)	851 (12.90%)	104 (5.90%)	132 (7.33%)	
Education, *N* (%)					< 0.001
Less than high school	3379 (22.88%)	1940 (29.40%)	540 (30.61%)	621 (34.46%)	
High school diploma	3324 (22.51%)	1441 (21.84%)	445 (25.23%)	471 (26.14%)	
More than high school	8067 (54.62%)	3217 (48.76%)	779 (44.16%)	710 (39.40%)	
Marital status, *N* (%)					< 0.001
Married or living with partner	9612 (65.08%)	4182 (63.38%)	970 (54.99%)	966 (53.61%)	
Single	5158 (34.92%)	2416 (36.62%)	794 (45.01%)	836 (46.39%)	—
Drinking status, *N* (%)					< 0.001
Nondrinkers	9292 (62.91%)	3763 (57.03%)	1063 (60.26%)	1011 (56.10%)	
Drinkers	4251 (28.78%)	2345 (35.54%)	568 (32.20%)	665 (36.90%)	
Missing	1227 (8.31%)	490 (7.43%)	133 (7.54%)	126 (6.99%)	
Smoking status, *N* (%)					< 0.001
Nonsmokers	6716 (45.47%)	2981 (45.18%)	1094 (62.02%)	1084 (60.16%)	
Smokers	8054 (54.53%)	3617 (54.82%)	670 (37.98%)	718 (39.84%)	
Physical activity, *N* (%)					< 0.001
Less active	5784 (39.16%)	1772 (26.86%)	885 (50.17%)	735 (40.79%)	
Active	6481 (43.88%)	1296 (19.64%)	599 (33.96%)	380 (21.09%)	
Missing	2505 (16.96%)	3530 (53.50%)	280 (15.87%)	687 (38.12%)	
BMI (kg/m^2^)	28.62 ± 6.41	31.08 ± 7.25	28.68 ± 6.29	32.19 ± 7.74	< 0.001
BMI categorized (kg/m^2^)					< 0.001
< 25	4365 (29.55%)	1252 (18.98%)	514 (29.14%)	266 (14.76%)	
≥ 25, < 30	5350 (36.22%)	2071 (31.39%)	631 (35.77%)	514 (28.52%)	—
≥ 30	5055 (34.22%)	3275 (49.64%)	619 (35.09%)	1022 (56.71%)	
Low family income, *N* (%)					< 0.001
No	12,182 (82.48%)	5245 (79.49%)	1371 (77.72%)	1352 (75.03%)	
Yes	2588 (17.52%)	1353 (20.51%)	393 (22.28%)	450 (24.97%)	
Hypertension, *N* (%)					< 0.001
No	8399 (56.87%)	2656 (40.25%)	432 (24.49%)	297 (16.48%)	
Yes	6371 (43.13%)	3942 (59.75%)	1332 (75.51%)	1505 (83.52%)	
Dyslipidemia, *N* (%)					< 0.001
No	3747 (25.37%)	1107 (16.78%)	335 (18.99%)	241 (13.37%)	
Yes	11,023 (74.63%)	5491 (83.22%)	1429 (81.01%)	1561 (86.63%)	

Abbreviations: BMI, body mass index; CVD, cardiovascular disease; PSM, propensity score matching.

⁣^∗^For continuous variables, *p* value was by survey-weighted linear regression; for categorical variables, *p* value was by survey-weighted chi-square test.

**Table 3:** Participants with diabetic and pre-existing evidence of CVD at baseline in 24,934 participants after PSM.

	**Total**	**No pre-existing CVD**		**Pre-existing CVD**		**p** **value**⁣^∗^
		**Nondiabetic**	**Diabetic**	**Nondiabetic**	**Diabetic**	
*N*	17,320	7463	6406	1715	1736	
All-cause mortality, *N* (%)						< 0.001
No	14,443 (83.39%)	6463 (86.60%)	5714 (89.20%)	1125 (65.60%)	1141 (65.73%)	
Yes	2877 (16.61%)	1000 (13.40%)	692 (10.80%)	590 (34.40%)	595 (34.27%)	
Cardiovascular mortality, *N* (%)						< 0.001
No	16,398 (94.68%)	7179 (96.19%)	6228 (97.22%)	1485 (86.59%)	1506 (86.75%)	
Yes	922 (5.32%)	284 (3.81%)	178 (2.78%)	230 (13.41%)	230 (13.25%)	
Follow-up (years)	6.93 ± 3.84	8.08 ± 3.86	5.97 ± 3.44	6.87 ± 4.01	5.63 ± 3.52	< 0.001
Age (years)	59.81 ± 13.24	58.47 ± 13.36	57.60 ± 12.64	66.55 ± 12.54	67.05 ± 10.73	< 0.001
Age categorical (years)						
< 65	10,645 (61.46%)	4884 (65.44%)	4403 (68.73%)	684 (39.88%)	674 (38.82%)	< 0.001
≥ 65	6675 (38.54%)	2579 (34.56%)	2003 (31.27%)	1031 (60.12%)	1062 (61.18%)	
Sex, *N* (%)						< 0.001
Man	5028 (29.03%)	2099 (28.13%)	1477 (23.06%)	818 (47.70%)	634 (36.52%)	
Woman	12,292 (70.97%)	5364 (71.87%)	4929 (76.94%)	897 (52.30%)	1102 (63.48%)	
Race/ethnicity, *N* (%)						< 0.001
Mexican American	2423 (13.99%)	958 (12.84%)	1146 (17.89%)	123 (7.17%)	196 (11.29%)	
Other Hispanic	1620 (9.35%)	644 (8.63%)	733 (11.44%)	92 (5.36%)	151 (8.70%)	
Non-Hispanic White	7591 (43.83%)	3539 (47.42%)	2177 (33.98%)	1045 (60.93%)	830 (47.81%)	
Non-Hispanic Black	3906 (22.55%)	1617 (21.67%)	1504 (23.48%)	352 (20.52%)	433 (24.94%)	
Other race	1780 (10.28%)	705 (9.45%)	846 (13.21%)	103 (6.01%)	126 (7.26%)	
Education, *N* (%)						< 0.001
Less than high school	4905 (28.32%)	1975 (26.46%)	1832 (28.60%)	517 (30.15%)	581 (33.47%)	
High school diploma	4035 (23.30%)	1734 (23.23%)	1409 (22.00%)	436 (25.42%)	456 (26.27%)	
More than high school	8380 (48.38%)	3754 (50.30%)	3165 (49.41%)	762 (44.43%)	699 (40.26%)	
Marital status, *N* (%)						< 0.001
Married or living with partner	10,629 (61.37%)	4686 (62.79%)	4056 (63.32%)	949 (55.34%)	938 (54.03%)	
Single	6691 (38.63%)	2777 (37.21%)	2350 (36.68%)	766 (44.66%)	798 (45.97%)	—
Drinking status, *N* (%)						0.002
Nondrinkers	10,135 (58.52%)	4459 (59.75%)	3673 (57.34%)	1026 (59.83%)	977 (56.28%)	
Drinkers	5878 (33.94%)	2425 (32.49%)	2252 (35.15%)	559 (32.59%)	642 (36.98%)	
Missing	1307 (7.55%)	579 (7.76%)	481 (7.51%)	130 (7.58%)	117 (6.74%)	
Smoking status, *N* (%)						< 0.001
Nonsmokers	8533 (49.27%)	3546 (47.51%)	2915 (45.50%)	1047 (61.05%)	1025 (59.04%)	
Smokers	8787 (50.73%)	3917 (52.49%)	3491 (54.50%)	668 (38.95%)	711 (40.96%)	
Physical activity, *N* (%)						< 0.001
Less active	6347 (36.65%)	3088 (41.38%)	1697 (26.49%)	854 (49.80%)	708 (40.78%)	
Active	5105 (29.47%)	2884 (38.64%)	1266 (19.76%)	584 (34.05%)	371 (21.37%)	
Missing	5868 (33.88%)	1491 (19.98%)	3443 (53.75%)	277 (16.15%)	657 (37.85%)	
BMI (kg/m^2^)	30.42 ± 7.06	29.95 ± 6.73	30.96 ± 7.26	28.70 ± 6.35	32.11 ± 7.83	< 0.001
BMI categorized (kg/m^2^)						< 0.001
< 25	3595 (20.76%)	1571 (21.05%)	1252 (19.54%)	506 (29.50%)	266 (15.32%)	
≥ 25, < 30	5664 (32.70%)	2491 (33.38%)	2063 (32.20%)	600 (34.99%)	510 (29.38%)	—
≥ 30	8061 (46.54%)	3401 (45.57%)	3091 (48.25%)	609 (35.51%)	960 (55.30%)	
Low family income, *N* (%)						< 0.001
No	13,789 (79.61%)	6032 (80.83%)	5104 (79.68%)	1333 (77.73%)	1320 (76.04%)	
Yes	3531 (20.39%)	1431 (19.17%)	1302 (20.32%)	382 (22.27%)	416 (23.96%)	
Hypertension, *N* (%)						< 0.001
No	6483 (37.43%)	3103 (41.58%)	2652 (41.40%)	432 (25.19%)	296 (17.05%)	
Yes	10,837 (62.57%)	4360 (58.42%)	3754 (58.60%)	1283 (74.81%)	1440 (82.95%)	
Dyslipidemia, *N* (%)						< 0.001
No	3020 (17.44%)	1346 (18.04%)	1103 (17.22%)	333 (19.42%)	238 (13.71%)	
Yes	14,300 (82.56%)	6117 (81.96%)	5303 (82.78%)	1382 (80.58%)	1498 (86.29%)	

Abbreviations: BMI, body mass index; CVD, cardiovascular disease; PSM, propensity score matching.

⁣^∗^For continuous variables, *p* value was by survey-weighted linear regression; for categorical variables, *p* value was by survey-weighted chi-square test.

**Table 4:** Incidence and Cox model for all-cause and cardiovascular mortality before PSM.

		**Incidence^#^**	**Crude model**	**Model 1**	**Model 2**	**Model 3**
All-cause mortality		18.85				
CVD^−^	DM^−^	12.22	Ref.	Ref.	Ref.	Ref.
	DM^+^	18.35	1.68 (1.53, 1.84)⁣^∗∗∗^	1.35 (1.23, 1.48)⁣^∗∗∗^	1.38 (1.25, 1.51)⁣^∗∗∗^	1.34 (1.22, 1.47)⁣^∗∗∗^
CVD^+^	DM^−^	51.75	Ref.	Ref.	Ref.	Ref.
	DM^+^	61.37	1.25 (1.12, 1.40)⁣^∗∗∗^	1.35 (1.20, 1.51)⁣^∗∗∗^	1.43 (1.27, 1.61)⁣^∗∗∗^	1.38 (1.22, 1.55)⁣^∗∗∗^
DM^−^	CVD^−^		Ref.	Ref.	Ref.	Ref.
	CVD^+^		4.39 (4.00, 4.82)⁣^∗∗∗^	1.76 (1.59, 1.94)⁣^∗∗∗^	1.67 (1.51, 1.84)⁣^∗∗∗^	1.65 (1.49, 1.82)⁣^∗∗∗^
DM^−^	CVD^−^		Ref.	Ref.	Ref.	Ref.
	CVD^+^		3.35 (3.01, 3.73)⁣^∗∗∗^	2.04 (1.82, 2.28)⁣^∗∗∗^	1.95 (1.74, 2.19)⁣^∗∗∗^	1.92 (1.72, 2.15)⁣^∗∗∗^
Cardiovascular mortality		5.74				
CVD^−^	DM^−^	3.10	Ref.	Ref.	Ref.	Ref.
	DM^+^	4.78	1.77 (1.48, 2.11)⁣^∗∗∗^	1.38 (1.15, 1.65)⁣^∗∗∗^	1.32 (1.10, 1.59)⁣^∗∗^	1.29 (1.07, 1.55)⁣^∗∗^
CVD^+^	DM^−^	20.37	Ref.	Ref.	Ref.	Ref.
	DM^+^	23.88	1.22 (1.02, 1.46)⁣^∗^	1.38 (1.15, 1.65)⁣^∗∗∗^	1.44 (1.19, 1.74)⁣^∗∗∗^	1.41 (1.16, 1.70)⁣^∗∗∗^
DM^−^	CVD^−^		Ref.	Ref.	Ref.	Ref.
	CVD^+^		6.81 (5.80, 8.00)⁣^∗∗∗^	2.40 (2.03, 2.84)⁣^∗∗∗^	2.28 (1.92, 2.70)⁣^∗∗∗^	2.25 (1.90, 2.67)⁣^∗∗∗^
DM^−^	CVD^−^		Ref.	Ref.	Ref.	Ref.
	CVD^+^		5.01 (4.14, 6.06)⁣^∗∗∗^	2.97 (2.44, 3.61)⁣^∗∗∗^	2.90 (2.37, 3.54)⁣^∗∗∗^	2.87 (2.35, 3.51)⁣^∗∗∗^

*Note:* DM^−^, nondiabetic participants; DM^+^, diabetic participants; CVD^−^, without pre-existing CVD; CVD^+^, with pre-existing CVD. Crude model: none. Model 1: sex, age, and race/ethnicity. Model 2: sex, age, race/ethnicity, education level, marital status, smoking status, low income, hypertension, and dyslipidemia. Model 3: sex, age, race/ethnicity, education level, marital status, smoking status, drinker, low income, physical activity, hypertension, and dyslipidemia.

^#^Per 1000 person-years. ⁣^∗^*p* < 0.05; ⁣^∗∗^< 0.01; ⁣^∗∗∗^< 0.001.

**Table 5:** Impact of diabetes and pre-existing CVD on all-cause and cardiovascular mortality risks before PSM.

		**Crude model**	**Model 1**	**Model 2**	**Model 3**
All-cause mortality					
CVD^−^	DM^−^	Ref.	Ref.	Ref.	Ref.
	DM^+^	1.66 (1.52, 1.81)⁣^∗∗∗^	1.34 (1.22, 1.46)⁣^∗∗∗^	1.38 (1.26, 1.51)⁣^∗∗∗^	1.34 (1.22, 1.47)⁣^∗∗∗^
CVD^+^	DM^−^	4.41 (4.01, 4.84)⁣^∗∗∗^	1.83 (1.66, 2.01)⁣^∗∗∗^	1.73 (1.57, 1.91)⁣^∗∗∗^	1.72 (1.56, 1.89)⁣^∗∗∗^
	DM^+^	5.58 (5.08, 6.13)⁣^∗∗∗^	2.59 (2.35, 2.85)⁣^∗∗∗^	2.53 (2.29, 2.80)⁣^∗∗∗^	2.42 (2.19, 2.68)⁣^∗∗∗^
*p* interaction		< 0.001	0.433	0.429	0.496
RERI		0.52 (0.07, 1.11)	0.41 (0.12, 0.69)	0.40 (0.13, 0.67)	0.39 (0.11, 0.67)
AP (%)		9.26 (−1.32, 18.54)	16.05 (5.15, 25.60)	16.13 (5.28, 25.42)	15.81 (4.82, 25.06)
SI		1.12 (0.98, 1.27)	1.36 (1.08, 1.67)	1.40 (1.08, 1.70)	1.36 (1.07, 1.69)
Cardiovascular mortality					
CVD^−^	DM^−^	Ref.	Ref.	Ref.	Ref.
	DM^+^	1.71 (1.44, 2.04)⁣^∗∗∗^	1.36 (1.14, 1.62)⁣^∗∗∗^	1.35 (1.13, 1.62)⁣^∗∗^	1.32 (1.10, 1.58)⁣^∗∗^
CVD^+^	DM^−^	6.86 (5.84, 8.06)⁣^∗∗∗^	2.58 (2.19, 3.04)⁣^∗∗∗^	2.44 (2.07, 2.88)⁣^∗∗∗^	2.42 (2.05, 2.87)⁣^∗∗∗^
	DM^+^	8.61 (7.32, 10.13)⁣^∗∗∗^	3.70 (3.13, 4.37)⁣^∗∗∗^	3.50 (2.94, 4.15)⁣^∗∗∗^	3.37 (2.83, 4.02)⁣^∗∗∗^
*p* interaction		0.015	0.662	0.616	0.681
RERI		1.04 (−0.34, 2.46)	0.74 (0.12, 1.32)	0.64 (0.06, 1.23)	0.65 (0.06, 1.25)
AP (%)		12.04 (−4.30, 25.84)	20.10 (3.65, 33.70)	18.73 (1.85, 32.56)	18.78 (1.96, 32.83)
SI		1.16 (0.94, 1.41)	1.38 (1.04, 1.82)	1.36 (1.01, 1.84)	1.36 (1.02, 2.84)

*Note:* DM^−^, nondiabetic participants; DM^−^, diabetic participants; CVD^−^, without pre-existing CVD; CVD^+^, with pre-existing CVD. Crude model: none. Model 1: sex, age, and race/ethnicity. Model 2: sex, age, race/ethnicity, education level, marital status, smoking status, low income, hypertension, and dyslipidemia. Model 3: sex, age, race/ethnicity, education level, marital status, smoking status, drinker, low income, physical activity, hypertension, and dyslipidemia.

Abbreviations: AP, attributable proportion; CVD, cardiovascular disease; PSM, propensity score matching; RERI, relative excess risk due to interaction; SI, synergy index.

⁣^∗∗^< 0.01; ⁣^∗∗∗^< 0.001.

**Table 6:** Impact of diabetes and pre-existing CVD on all-cause and cardiovascular mortality risks after PSM.

		**Crude model**	**Model 1**	**Model 2**	**Model 3**
All-cause mortality					
CVD^−^	DM^−^	Ref.	Ref.	Ref.	Ref.
	DM^+^	1.21 (1.09, 1.33)⁣^∗∗^	1.35 (1.23, 1.49)⁣^∗∗^	1.38 (1.25, 1.53)⁣^∗∗^	1.35 (1.22, 1.49)⁣^∗∗^
CVD^+^	DM^−^	3.12 (2.82, 3.45)⁣^∗∗^	1.83 (1.65, 2.03)⁣^∗∗^	1.75 (1.58, 1.94)⁣^∗∗^	1.74 (1.57, 1.93)⁣^∗∗^
	DM^+^	4.07 (3.68, 4.51)⁣^∗∗^	2.66 (2.39, 2.95)⁣^∗∗^	2.65 (2.39, 2.94)⁣^∗∗^	2.55 (2.29, 2.83)⁣^∗∗^
*p* interaction		< 0.001	0.200	0.293	0.374
RERI		0.74	0.48	0.52	0.46
AP (%)		18.18	18.05	19.62	18.04
SI		1.32	1.41	1.46	1.42
Cardiovascular mortality					
CVD^−^	DM^−^	Ref.	Ref.	Ref.	Ref.
	DM^+^	1.09 (0.90, 1.32)	1.23 (1.02, 1.49)⁣^∗^	1.25 (1.03, 1.51)⁣^∗^	1.22 (1.01, 1.48)⁣^∗^
CVD^+^	DM^−^	4.29 (3.60, 5.10)⁣^∗∗^	2.42 (2.03, 2.89)⁣^∗∗^	2.33 (1.95, 2.78)⁣^∗∗^	2.31 (1.94, 2.76)⁣^∗∗^
	DM^+^	5.55 (4.66, 6.61)⁣^∗∗^	3.49 (2.92, 4.17)⁣^∗∗^	3.43 (2.87, 4.11)⁣^∗∗^	3.34 (2.78, 4.00)⁣^∗∗^
*p* interaction		0.015	0.293	0.200	0.242
RERI		1.17	0.84	0.85	0.81
AP (%)		21.08	24.07	24.78	24.25
SI		1.35	1.51	1.54	1.53

*Note:* DM^−^, nondiabetic participants; DM^−^, diabetic participants; CVD^−^, without pre-existing CVD; CVD^+^, with pre-existing CVD. Crude model: none. Model 1: sex, age, and race/ethnicity. Model 2: sex, age, race/ethnicity, education level, marital status, smoking status, low income, hypertension, and dyslipidemia. Model 3: sex, age, race/ethnicity, education level, marital status, smoking status, drinker, low income, physical activity, hypertension, and dyslipidemia.

Abbreviations: AP, attributable proportion; CVD, cardiovascular disease; PSM, propensity score matching; RERI, relative excess risk due to interaction; SI, synergy index.

⁣^∗^< 0.05; ⁣^∗∗^< 0.01.

## Data Availability

All data in the article were obtained from the NHANES database (https://wwwn.cdc.gov/nchs/nhanes/).

## Supporting Information

Supporting Information Additional supporting information can be found online in the Supporting Information section. Table S1 Characteristics of adults according to cardiovascular mortality before PSM. Table S2 Characteristics of adults according to diabetes before PSM. Table S3 Characteristics of adults according to pre-existing CVD before PSM. Table S4 Selected baseline characteristics in propensity score matched groups. Table S5 Characteristics of 24,934 participants according to all-cause mortality after PSM. Table S6 Characteristics of adults according to cardiovascular mortality before PSM. Table S7 Characteristics of 17,320 participants according to diabetes after PSM. Table S8 Characteristics of 17,320 participants according to pre-existing CVD after PSM. Table S9 Subgroup analysis on the effects of the diabetes and pre-existing CVD on the risk of all-cause mortality using nondiabetic participants without pre-existing CVD as reference. Table S10 Subgroup analysis on the effects of the diabetes and pre-existing CVD on the risks of cardiovascular mortality before PSM using nondiabetic participants without pre-existing CVD as reference.
