# Supplementary material for: Bidirectional Mediation and Synergistic Mortality Risks in Diabetes and Cardiovascular Disease: Evidence From NHANES 2005–2018
Source: J Diabetes Res. 2025 Nov 10;2025:8517492. doi: 10.1155/jdr/8517492 (PMC12623085; doi:10.1155/jdr/8517492)
Supplement: Supporting Information — Additional supporting information can be found online in the Supporting Information section. Table S1 Characteristics of adults according to cardiovascular mortality before PSM. Table S2 Characteristics of adults according to diabetes before PSM. Table S3 Characteristics of adults according to pre-existing CVD before PSM. Table S4 Selected baseline characteristics in propensity score matched groups. Table S5 Characteristics of 24,934 participants according to all-cause mortality after PSM. Table S6 Characteristics of adults according to cardiovascular mortality before PSM. Table S7 Characteristics of 17,320 participants according to diabetes after PSM. Table S8 Characteristics of 17,320 participants according to pre-existing CVD after PSM. Table S9 Subgroup analysis on the effects of the diabetes and pre-existing CVD on the risk of all-cause mortality using nondiabetic participants without pre-existing CVD as reference. Table S10 Subgroup analysis on the effects of the diabetes and pre-existing CVD on the risks of cardiovascular mortality before PSM using nondiabetic participants without pre-existing CVD as reference. [file 8517492.f1.docx]

**Table S1** Characteristics of adults according to cardiovascular mortality before PSM

|  | **Survival** | | **Morality** | | **P-value*** | |  |
| --- | --- | --- | --- | --- | --- | --- | --- |
| **N** | 23,879 | | 1,055 | |  | |  |
| **Follow-up (years)** | 7.45 ± 3.93 | | 5.56 ± 3.41 | | < 0.001 | |  |
| **Age (years)** | 56.36 ± 13.36 | | 72.37 ± 10.38 | | < 0.001 | |  |
| **Age categorical** |  | |  | | < 0.001 | |  |
| **< 65** | 16901 (70.78%) | | 218 (20.66%) | |  | |  |
| **≥ 65** | 6978 (29.22%) | | 837 (79.34%) | |  | |  |
| **Sex, N (%)** |  | |  | | < 0.001 | |  |
| **Man** | 8269 (34.63%) | | 504 (47.77%) | |  | |  |
| **Woman** | 15610 (65.37%) | | 551 (52.23%) | |  | |  |
| **Race/Ethnicity, N (%)** |  | |  | | < 0.001 | |  |
| **Mexican American** | 3435 (14.39%) | | 74 (7.01%) | |  | |  |
| **Other Hispanic** | 2201 (9.22%) | | 56 (5.31%) | |  | |  |
| **Non-Hispanic White** | 10514 (44.03%) | | 670 (63.51%) | |  | |  |
| **Non-Hispanic Black** | 5115 (21.42%) | | 220 (20.85%) | |  | |  |
| **Other Race** | 2614 (10.95%) | | 35 (3.32%) | |  | |  |
| **E****ducation, N (%)** |  | |  | | < 0.001 | |  |
| **Less than high school** | 6093 (25.52%) | | 387 (36.68%) | |  | |  |
| **High school diploma** | 5406 (22.64%) | | 275 (26.07%) | |  | |  |
| **More than high school** | 12380 (51.84%) | | 393 (37.25%) | |  | |  |
| **M****arital status, N (%)** |  | |  | | < 0.001 | |  |
| **Married or living with partner** | | 15234 (63.80%) | | 496 (47.01%) | |  | |
| **Single** | | 8645 (36.20%) | | 559 (52.99%) | | - | |
| **D****rinking status, N (%)** |  | |  | | < 0.001 | |  |
| **Nondrinkers** | 14511 (60.77%) | | 618 (58.58%) | |  | |  |
| **Drinkers** | 7468 (31.27%) | | 361 (34.22%) | |  | |  |
| **Missing** | 1900 (7.96%) | | 76 (7.20%) | |  | |  |
| **Smoking status, N (%)** |  | |  | | < 0.001 | |  |
| **Non-smokers** | 11298 (47.31%) | | 577 (54.69%) | |  | |  |
| **Smokers** | 12581 (52.69%) | | 478 (45.31%) | |  | |  |
| **Physical activity, N (%)** |  | |  | | < 0.001 | |  |
| **Less active** | 8566 (35.87%) | | 610 (57.82%) | |  | |  |
| **Active** | 8498 (35.59%) | | 258 (24.45%) | |  | |  |
| **Missing** | 6815 (28.54%) | | 187 (17.73%) | |  | |  |
| **BMI (kg/m^2^)** | 29.55 ± 6.85 | | 29.23 ± 7.11 | | 0.358 | |  |
| **BMI categorical (kg/m^2^)** |  | |  | | 0.523 | |  |
| **< 25** | 6111 (25.59%) | | 286 (27.11%) | |  | |  |
| **≥ 25, < 30** | 8196 (34.32%) | | 370 (35.07%) | |  | |  |
| **≥ 30** | 9572 (40.09%) | | 399 (37.82%) | |  | |  |
| **Low family income, N (%)** |  | |  | | < 0.001 | |  |
| **Yes** | 19314 (80.88%) | | 836 (79.24%) | |  | |  |
| **No** | 4565 (19.12%) | | 219 (20.76%) | |  | |  |
| **Hypertension, N (%)** |  | |  | | < 0.001 | |  |
| **Yes** | 11562 (48.42%) | | 222 (21.04%) | |  | |  |
| **No** | 12317 (51.58%) | | 833 (78.96%) | |  | |  |
| **Dyslipidemia, N (%)** |  | |  | | 0.101 | |  |
| **Yes** | 5213 (21.83%) | | 217 (20.57%) | |  | |  |
| **No** | 18666 (78.17%) | | 838 (79.43%) | |  | |  |
| **Pre-existing CVD, N (%)** |  | |  | | < 0.001 | |  |
| **No** | 20801 (87.11%) | | 567 (53.74%) | |  | |  |
| **Yes** | 3078 (12.89%) | | 488 (46.26%) | |  | |  |
| **Diabetes, N (%)** |  | |  | | < 0.001 | |  |
| **No** | 15910 (66.63%) | | 624 (59.15%) | |  | |  |
| **Yes** | 7969 (33.37%) | | 431 (40.85%) | |  | |  |

Notes: BMI, body mass index; CVD, cardiovascular disease; PSM, propensity score matching

* For continuous variables: P-value was by survey-weighted linear regression; For categorical variables: P-value was by survey-weighted Chi-square test

**Table S2** Characteristics of adults according to diabetes before PSM

|  | **Non-diabetes** | **Diabetes** | **P-value^*^** |
| --- | --- | --- | --- |
| **N** | 16534 | 8400 |  |
| **All-cause mortality, N (%)** |  |  | < 0.001 |
| **No** | 14419 (87.21%) | 7053 (83.96%) |  |
| **Yes** | 2115 (12.79%) | 1347 (16.04%) |  |
| **Cardiovascular mortality, N (%)** |  |  | < 0.001 |
| **No** | 15910 (96.23%) | 7969 (94.87%) |  |
| **Yes** | 624 (3.77%) | 431 (5.13%) |  |
| **Follow-up (years)** | 8.11 ± 3.94 | 5.91 ± 3.46 | < 0.001 |
| **Age (years)** | 55.54 ± 13.75 | 59.98 ± 12.91 | < 0.001 |
| **Age categorical (years)** |  |  |  |
| **< 65** | 14770 (89.33%) | 6598 (78.55%) | < 0.001 |
| **≥ 65** | 1764 (10.67%) | 1802 (21.45%) |  |
| **Sex, N (%)** |  |  | < 0.001 |
| **Man** | 6643 (40.18%) | 2130 (25.36%) |  |
| **Woman** | 9891 (59.82%) | 6270 (74.64%) |  |
| **Race/Ethnicity, N (%)** |  |  | < 0.001 |
| **Mexican American** | 2111 (12.77%) | 1398 (16.64%) |  |
| **Other Hispanic** | 1346 (8.14%) | 911 (10.85%) |  |
| **Non-Hispanic White** | 8077 (48.85%) | 3107 (36.99%) |  |
| **Non-Hispanic Black** | 3334 (20.16%) | 2001 (23.82%) |  |
| **Other Race** | 1666 (10.08%) | 983 (11.70%) |  |
| **Education, N (%)** |  |  | < 0.001 |
| **Less than high school** | 3919 (23.70%) | 2561 (30.49%) |  |
| **High school diploma** | 3769 (22.80%) | 1912 (22.76%) |  |
| **More than high school** | 8846 (53.50%) | 3927 (46.75%) |  |
| **Marital status, N (%)** |  |  | 0.043 |
| **Married or living with partner** | 10582 (64.00%) | 5148 (61.29%) |  |
| **Single** | 5952 (36.00%) | 3252 (38.71%) | - |
| **Drinking status, N (%)** |  |  | < 0.001 |
| **Nondrinkers** | 10355 (62.63%) | 4774 (56.83%) |  |
| **Drinkers** | 4819 (29.15%) | 3010 (35.83%) |  |
| **Missing** | 1360 (8.23%) | 616 (7.33%) |  |
| **Smoking status, N (%)** |  |  | < 0.001 |
| **Non-smokers** | 7810 (47.24%) | 4065 (48.39%) |  |
| **Smokers** | 8724 (52.76%) | 4335 (51.61%) |  |
| **Physical activity, N (%)** |  |  | < 0.001 |
| **Less active** | 6669 (40.34%) | 2507 (29.85%) |  |
| **Active** | 7080 (42.82%) | 1676 (19.95%) |  |
| **Missing** | 2785 (16.84%) | 4217 (50.20%) |  |
| **BMI (kg/m^2^)** | 28.63 ± 6.39 | 31.32 ± 7.37 | < 0.001 |
| **BMI categorical (kg/m^2^)** |  |  | < 0.001 |
| **< 25** | 4879 (29.51%) | 1518 (18.07%) |  |
| **≥ 25, < 30** | 5981 (36.17%) | 2585 (30.77%) | - |
| **≥ 30** | 5674 (34.32%) | 4297 (51.15%) |  |
| **Low family income, N (%)** |  |  | 0.001 |
| **Yes** | 13553 (81.97%) | 6597 (78.54%) |  |
| **No** | 2981 (18.03%) | 1803 (21.46%) |  |
| **Hypertension, N (%)** |  |  | < 0.001 |
| **No** | 8831 (53.41%) | 2953 (35.15%) |  |
| **Yes** | 7703 (46.59%) | 5447 (64.85%) |  |
| **Dyslipidemia, N (%)** |  |  | < 0.001 |
| **No** | 4082 (24.69%) | 1348 (16.05%) |  |
| **Yes** | 12452 (75.31%) | 7052 (83.95%) |  |
| **Pre-existing CVD, N (%)** |  |  | < 0.001 |
| **No** | 14770 (89.33%) | 6598 (78.55%) |  |
| **Yes** | 1764 (10.67%) | 1802 (21.45%) |  |

Notes: BMI, body mass index; CVD, cardiovascular disease; PSM, propensity score matching

* For continuous variables: P-value was by survey-weighted linear regression; For categorical variables: P-value was by survey-weighted Chi-square test

**Table S3** Characteristics of adults according to pre-existing CVD before PSM

|  | **Without**  **pre-existing CVD** | **Pre-existing CVD** | **P-value*** |
| --- | --- | --- | --- |
| **N** | 21368 | 3566 |  |
| **All-cause mortality, N (%)** |  |  | < 0.001 |
| **No** | 19153 (89.63%) | 2319 (65.03%) |  |
| **Yes** | 2215 (10.37%) | 1247 (34.97%) |  |
| **Cardiovascular mortality, N (%)** |  |  | < 0.001 |
| **No** | 20801 (97.35%) | 3078 (86.32%) |  |
| **Yes** | 567 (2.65%) | 488 (13.68%) |  |
| **Follow-up (years)** | 7.56 ± 3.91 | 6.23 ± 3.81 | < 0.001 |
| **Age (years)** | 55.35 ± 13.20 | 67.14 ± 11.66 | < 0.001 |
| **Age categorical (years)** |  |  |  |
| **< 65** | 15759 (73.75%) | 1360 (38.14%) | < 0.001 |
| **≥ 65** | 5609 (26.25%) | 2206 (61.86%) |  |
| **Sex, N (%)** |  |  | < 0.001 |
| **Man** | 7262 (33.99%) | 1511 (42.37%) |  |
| **Woman** | 14106 (66.01%) | 2055 (57.63%) |  |
| **Race/Ethnicity, N (%)** |  |  | < 0.001 |
| **Mexican American** | 3183 (14.90%) | 326 (9.14%) |  |
| **Other Hispanic** | 2007 (9.39%) | 250 (7.01%) |  |
| **Non-Hispanic White** | 9247 (43.27%) | 1937 (54.32%) |  |
| **Non-Hispanic Black** | 4518 (21.14%) | 817 (22.91%) |  |
| **Other Race** | 2413 (11.29%) | 236 (6.62%) |  |
| **Education, N (%)** |  |  | < 0.001 |
| **Less than high school** | 5319 (24.89%) | 1161 (32.56%) |  |
| **High school diploma** | 4765 (22.30%) | 916 (25.69%) |  |
| **More than high school** | 11284 (52.81%) | 1489 (41.76%) |  |
| **Marital status, N (%)** |  |  | < 0.001 |
| **Married or living with partner** | 13794 (64.55%) | 1936 (54.29%) |  |
| **Single** | 7574 (35.45%) | 1630 (45.71%) | - |
| **Drinking status, N (%)** |  |  | < 0.001 |
| **Nondrinkers** | 13055 (61.10%) | 2074 (58.16%) |  |
| **Drinkers** | 6596 (30.87%) | 1233 (34.58%) |  |
| **Missing** | 1717 (8.04%) | 259 (7.26%) |  |
| **Smoking status, N (%)** |  |  | < 0.001 |
| **Non-smokers** | 9697 (45.38%) | 2178 (61.08%) |  |
| **Smokers** | 11671 (54.62%) | 1388 (38.92%) |  |
| **Physical activity, N (%)** |  |  | < 0.001 |
| **Less active** | 7556 (35.36%) | 1620 (45.43%) |  |
| **Active** | 7777 (36.40%) | 979 (27.45%) |  |
| **Missing** | 6035 (28.24%) | 967 (27.12%) |  |
| **BMI (kg/m^2^)** |  |  | < 0.001 |
| **BMI categorized (kg/m^2^)** |  |  | < 0.001 |
| **< 25** | 5617 (26.29%) | 780 (21.87%) |  |
| **≥ 25, < 30** | 7421 (34.73%) | 1145 (32.11%) | - |
| **≥ 30** | 8330 (38.98%) | 1641 (46.02%) |  |
| **Low family income, N (%)** |  |  | < 0.001 |
| **Yes** | 17427 (81.56%) | 2723 (76.36%) |  |
| **No** | 3941 (18.44%) | 843 (23.64%) |  |
| **Hypertension, N (%)** |  |  | < 0.001 |
| **No** | 11055 (51.74%) | 729 (20.44%) |  |
| **Yes** | 10313 (48.26%) | 2837 (79.56%) |  |
| **Dyslipidemia, N (%)** |  |  | < 0.001 |
| **No** | 4854 (22.72%) | 576 (16.15%) |  |
| **Yes** | 16514 (77.28%) | 2990 (83.85%) |  |

Notes: BMI, body mass index; CVD, cardiovascular disease; PSM, propensity score matching

* For continuous variables: P-value was by survey-weighted linear regression; For categorical variables: P-value was by survey-weighted Chi-square test

**Table S4** Selected baseline characteristics in propensity-score matched groups

|  | **Pair 1 (n= 6,406)** | | **Pair 2 (n= 1,715)** | | **Pair 3 (n= 1,736)** | |
| --- | --- | --- | --- | --- | --- | --- |
|  | **DM ^-^ CVD ^-^** | **DM ^+^ CVD ^-^** | **DM ^-^ CVD ^-^** | **DM ^-^ CVD ^+^** | **DM ^-^ CVD ^-^** | **DM ^+^ CVD ^+^** |
| **N** | 6406 | 6406 | 1715 | 1715 | 1736 | 1736 |
| **Age (years)** | 57.59 ± 13.28 | 57.60 ± 12.64 | 66.79 ± 11.98 | 66.55 ± 12.54 | 67.03 ± 11.26 | 67.05 ± 10.73 |
| **Sex, N (%)** |  |  |  |  |  |  |
| **Man** | 1412 (22.04%) | 1477 (23.06%) | 806 (47.00%) | 818 (47.70%) | 617 (35.54%) | 634 (36.52%) |
| **Woman** | 4994 (77.96%) | 4929 (76.94%) | 909 (53.00%) | 897 (52.30%) | 1119 (64.46%) | 1102 (63.48%) |
| **Race/Ethnicity, N (%)** |  |  |  |  |  |  |
| **Mexican American** | 875 (13.66%) | 1146 (17.89%) | 129 (7.52%) | 123 (7.17%) | 147 (8.47%) | 196 (11.29%) |
| **Other Hispanic** | 578 (9.02%) | 733 (11.44%) | 105 (6.12%) | 92 (5.36%) | 121 (6.97%) | 151 (8.70%) |
| **Non-Hispanic White** | 2953 (46.10%) | 2177 (33.98%) | 1029 (60.00%) | 1045 (60.93%) | 937 (53.97%) | 830 (47.81%) |
| **Non-Hispanic Black** | 1376 (21.48%) | 1504 (23.48%) | 345 (20.12%) | 352 (20.52%) | 432 (24.88%) | 433 (24.94%) |
| **Other Race** | 624 (9.74%) | 846 (13.21%) | 107 (6.24%) | 103 (6.01%) | 99 (5.70%) | 126 (7.26%) |
| **Education, N (%)** |  |  |  |  |  |  |
| **Less than high school** | 1724 (26.91%) | 1832 (28.60%) | 457 (26.65%) | 517 (30.15%) | 533 (30.70%) | 581 (33.47%) |
| **High school diploma** | 1465 (22.87%) | 1409 (22.00%) | 433 (25.25%) | 436 (25.42%) | 464 (26.73%) | 456 (26.27%) |
| **More than high school** | 3217 (50.22%) | 3165 (49.41%) | 825 (48.10%) | 762 (44.43%) | 739 (42.57%) | 699 (40.26%) |
| **Marital status, N (%)** |  |  |  |  |  |  |
| **Married or living with partner** | 4076 (63.63%) | 4056 (63.32%) | 993 (57.90%) | 949 (55.34%) | 895 (51.56%) | 938 (54.03%) |
| **Single** | 2330 (36.37%) | 2350 (36.68%) | 722 (42.10%) | 766 (44.66%) | 841 (48.44%) | 798 (45.97%) |
| **Smoking status, N (%)** |  |  |  |  |  |  |
| **Non-smokers** | 2857 (44.60%) | 2915 (45.50%) | 1037 (60.47%) | 1047 (61.05%) | 1016 (58.53%) | 1025 (59.04%) |
| **Smokers** | 3549 (55.40%) | 3491 (54.50%) | 678 (39.53%) | 668 (38.95%) | 720 (41.47%) | 711 (40.96%) |
| **BMI (kg/m^2^)** | 30.31 ± 6.82 | 30.96 ± 7.26 | 28.82 ± 6.42 | 28.70 ± 6.35 | 30.80 ± 6.36 | 32.11 ± 7.83 |
| **BMI categorized** |  |  |  |  |  |  |
| **< 25** | 1231 (19.22%) | 1252 (19.54%) | 452 (26.36%) | 506 (29.50%) | 241 (13.88%) | 266 (15.32%) |
| **≥ 25, < 30** | 2070 (32.31%) | 2063 (32.20%) | 628 (36.62%) | 600 (34.99%) | 557 (32.09%) | 510 (29.38%) |
| **≥ 30** | 3105 (48.47%) | 3091 (48.25%) | 635 (37.03%) | 609 (35.51%) | 938 (54.03%) | 960 (55.30%) |
| **Low family income, N (%)** |  |  |  |  |  |  |
| **Yes** | 5176 (80.80%) | 5104 (79.68%) | 1390 (81.05%) | 1333 (77.73%) | 1347 (77.59%) | 1320 (76.04%) |
| **No** | 1230 (19.20%) | 1302 (20.32%) | 325 (18.95%) | 382 (22.27%) | 389 (22.41%) | 416 (23.96%) |
| **Hypertension, N (%)** |  |  |  |  |  |  |
| **Yes** | 2758 (43.05%) | 2652 (41.40%) | 422 (24.61%) | 432 (25.19%) | 283 (16.30%) | 296 (17.05%) |
| **No** | 3648 (56.95%) | 3754 (58.60%) | 1293 (75.39%) | 1283 (74.81%) | 1453 (83.70%) | 1440 (82.95%) |
| **Dyslipidemia, N (%)** |  |  |  |  |  |  |
| **Yes** | 1107 (17.28%) | 1103 (17.22%) | 294 (17.14%) | 333 (19.42%) | 216 (12.44%) | 238 (13.71%) |
| **No** | 5299 (82.72%) | 5303 (82.78%) | 1421 (82.86%) | 1382 (80.58%) | 1520 (87.56%) | 1498 (86.29%) |

**Table S5** Characteristics of 24,934 participants according to all-cause mortality after PSM

|  | **Survival** | | | **Morality** | | | | **P-value^*^** | | | | |  |  |
| --- | --- | --- | --- | --- | --- | --- | --- | --- | --- | --- | --- | --- | --- | --- |
| **N** | 21472 | | | 3462 | | | |  | | | | |  |  |
| **Participants, N (%)** |  | | |  | | | | | < 0.001 | | | | | |
| **Diabetes ^-^, Pre-existing CVD ^-^** | | | 6463 (44.75%) | | | 1000 (34.76%) | | | | |  | | |  |
| **Diabetes ^+^, Pre-existing CVD ^-^** | | | 5714 (39.56%) | | | 692 (24.05%) | | | | |  | | |  |
| **Diabetes ^-^, Pre-existing CVD ^+^** | | | 1125 (7.79%) | | | 590 (20.51%) | | | | |  | | |  |
| **Diabetes ^+^, Pre-existing CVD ^+^** | | | 1141 (7.90%) | | | 595 (20.68%) | | | | |  | | |  |
| **Follow-up (years)** | 7.22 ± 3.84 | | | 5.49 ± 3.45 | | | | | < 0.001 | | | | | |
| **Pre-existing CVD, N (%)** |  | | |  | | | | | < 0.001 | | | | | |
| **No** | 12177 (84.31%) | | | 1692 (58.81%) | | | | |  | | | | | |
| **Yes** | 2266 (15.69%) | | | 1185 (41.19%) | | | | |  | | | | | |
| **Diabetes, N (%)** |  | | |  | | | | | < 0.001 | | | | | |
| **No** | | 7588 (52.54%) | | | 1590 (55.27%) | | | | |  | | | |  |
| **Yes** | | 6855 (47.46%) | | | 1287 (44.73%) | | | | |  | | | |  |
| **Age (years)** | 57.55 ± 12.54 | | | 71.18 ± 10.56 | | | | < 0.001 | | | | |  |  |
| **Age categorical (years)** |  | | |  | | | | < 0.001 | | | | |  |  |
| **< 65** | 9943 (68.84%) | | | 702 (24.40%) | | | |  | | | | |  |  |
| **≥ 65** | 4500 (31.16%) | | | 2175 (75.60%) | | | |  | | | | |  |  |
| **Sex, N (%)** |  | | |  | | | | < 0.001 | | | | |  |  |
| **Man** | 3783 (26.19%) | | | 1245 (43.27%) | | | |  | | | | |  |  |
| **Woman** | 10660 (73.81%) | | | 1632 (56.73%) | | | |  | | | | |  |  |
| **Race/Ethnicity, N (%)** |  | | |  | | | | < 0.001 | | | | |  |  |
| **Mexican American** | 2191 (15.17%) | | | 232 (8.06%) | | | |  | | | | |  |  |
| **Other Hispanic** | 1470 (10.18%) | | | 150 (5.21%) | | | |  | | | | |  |  |
| **Non-Hispanic White** | 5804 (40.19%) | | | 1787 (62.11%) | | | |  | | | | |  |  |
| **Non-Hispanic Black** | 3315 (22.95%) | | | 591 (20.54%) | | | |  | | | | |  |  |
| **Other Race** | 1663 (11.51%) | | | 117 (4.07%) | | | |  | | | | |  |  |
| **Education, N (%)** |  | | |  | | | | < 0.001 | | | | |  |  |
| **Less than high school** | 3853 (26.68%) | | | 1052 (36.57%) | | | |  | | | | |  |  |
| **High school diploma** | 3274 (22.67%) | | | 761 (26.45%) | | | |  | | | | |  |  |
| **More than high school** | 7316 (50.65%) | | | 1064 (36.98%) | | | |  | | | | |  |  |
| **Marital status, N (%)** |  | | |  | | | | < 0.001 | | | | |  |  |
| **Married or living with partner** | 9209 (63.76%) | | | 1420 (49.36%) | | | |  | | | | |  |  |
| **Single** | 5234 (36.24%) | | | 1457 (50.64%) | | | | - | | | | |  |  |
| **Drinking status, N (%)** |  | | |  | | | | 0.252 | | | | |  |  |
| **Nondrinkers** | 8456 (58.55%) | | | 1679 (58.36%) | | | |  | | | | |  |  |
| **Drinkers** | 4918 (34.05%) | | | 960 (33.37%) | | | |  | | | | |  |  |
| **Missing** | 1069 (7.40%) | | | 238 (8.27%) | | | |  | | | | |  |  |
| **Smoking status, N (%)** |  | | |  | | | | < 0.001 | | | | |  |  |
| **Non-smokers** | 6794 (47.04%) | | | 1739 (60.44%) | | | |  | | | | |  |  |
| **Smokers** | 7649 (52.96%) | | | 1138 (39.56%) | | |  | | | | |  |  |  |
| **Physical activity, N (%)** |  | | |  | | | < 0.001 | | | | |  |  |  |
| **Less active** | 4717 (32.66%) | | | 1630 (56.66%) | | | |  | | | | |  |  |
| **Active** | 4449 (30.80%) | | | 656 (22.80%) | | | |  | | | | |  |  |
| **Missing** | 5277 (36.54%) | | | 591 (20.54%) | | | |  | | | | |  |  |
| **BMI (kg/m^2^)** | 30.67 ± 7.03 | | | 29.14 ± 7.08 | | | | < 0.001 | | | | |  |  |
| **BMI categorical (kg/m^2^)** |  | | |  | | | | < 0.001 | | | | |  |  |
| **< 25** | 2778 (19.23%) | | | 817 (28.40%) | | | |  | | | | |  |  |
| **≥ 25, < 30** | 4690 (32.47%) | | | 974 (33.85%) | | | | - | | | | |  |  |
| **≥ 30** | 6975 (48.29%) | | | 1086 (37.75%) | | | |  | | | | |  |  |
| **Low family income, N (%)** |  | | |  | | | | < 0.001 | | | | |  |  |
| **No** | 11546 (79.94%) | | | 2243 (77.96%) | | | |  | | | | |  |  |
| **Yes** | 2897 (20.06%) | | | 634 (22.04%) | | | |  | | | | |  |  |
| **Hypertension, N (%)** |  | | |  | | | | < 0.001 | | | | |  |  |
| **No** | 5840 (40.43%) | | | 643 (22.35%) | | | |  | | | | |  |  |
| **Yes** | 8603 (59.57%) | | | 2234 (77.65%) | | | |  | | | | |  |  |
| **Dyslipidemia, N (%)** |  | | |  | | | | < 0.001 | | | | |  |  |
| **No** | 2437 (16.87%) | | | 583 (20.26%) | | | |  | | | | |  |  |
| **Yes** | 12006 (83.13%) | | | 2294 (79.74%) | | | |  | | | | |  |  |

Notes: BMI, body mass index; CVD, cardiovascular disease; PSM, propensity score matching

* For continuous variables: P-value was by survey-weighted linear regression; For categorical variables: P-value was by survey-weighted Chi-square test

**Table S6** Characteristics of adults according to cardiovascular mortality before PSM

|  | **Survival** | **Morality** | **P-value*** |
| --- | --- | --- | --- |
| **N** | 16398 | 922 |  |
| **Follow-up (years)** | 7.01 ± 3.84 | 5.51 ± 3.44 | < 0.001 |
| **Participants, N (%)** |  |  | < 0.001 |
| **Diabetes ^-^, Pre-existing CVD ^-^** | 7179 (43.78%) | 284 (30.80%) |  |
| **Diabetes ^+^, Pre-existing CVD ^-^** | 6228 (37.98%) | 178 (19.31%) |  |
| **Diabetes ^-^, Pre-existing CVD ^+^** | 1485 (9.06%) | 230 (24.95%) |  |
| **Diabetes ^+^, Pre-existing CVD ^+^** | 1506 (9.18%) | 230 (24.95%) |  |
| **Follow-up (years)** | 7.01 ± 3.84 | 5.51 ± 3.44 | < 0.001 |
| **Pre-existing CVD, N (%)** |  |  | < 0.001 |
| **No** | 13407 (81.76%) | 462 (50.11%) |  |
| **Yes** | 2991 (18.24%) | 460 (49.89%) |  |
| **Diabetes, N (%)** |  |  | < 0.001 |
| **No** | 8664 (52.84%) | 514 (55.75%) |  |
| **Yes** | 7734 (47.16%) | 408 (44.25%) |  |
| **Age (years)** |  |  | < 0.001 |
| **Age categorical** |  |  | < 0.001 |
| **< 65** | 10456 (63.76%) | 189 (20.50%) |  |
| **≥ 65** | 5942 (36.24%) | 733 (79.50%) |  |
| **Sex, N (%)** |  |  | < 0.001 |
| **Man** | 4605 (28.08%) | 423 (45.88%) |  |
| **Woman** | 11793 (71.92%) | 499 (54.12%) |  |
| **Race/Ethnicity, N (%)** |  |  | < 0.001 |
| **Mexican American** | 2354 (14.36%) | 69 (7.48%) |  |
| **Other Hispanic** | 1569 (9.57%) | 51 (5.53%) |  |
| **Non-Hispanic White** | 7015 (42.78%) | 576 (62.47%) |  |
| **Non-Hispanic Black** | 3712 (22.64%) | 194 (21.04%) |  |
| **Other Race** | 1748 (10.66%) | 32 (3.47%) |  |
| **Education, N (%)** |  |  | < 0.001 |
| **Less than high school** | 4559 (27.80%) | 346 (37.53%) |  |
| **High school diploma** | 3799 (23.17%) | 236 (25.60%) |  |
| **More than high school** | 8040 (49.03%) | 340 (36.88%) |  |
| **Marital status, N (%)** |  |  | < 0.001 |
| **Married or living with partner** | 10193 (62.16%) | 436 (47.29%) |  |
| **Single** | 6205 (37.84%) | 486 (52.71%) | - |
| **Drinking status, N (%)** |  |  | < 0.001 |
| **Nondrinkers** | 9598 (58.53%) | 537 (58.24%) |  |
| **Drinkers** | 5560 (33.91%) | 318 (34.49%) |  |
| **Missing** | 1240 (7.56%) | 67 (7.27%) |  |
| **Smoking status, N (%)** |  |  | 0.003 |
| **Non-smokers** | 8035 (49.00%) | 498 (54.01%) |  |
| **Smokers** | 8363 (51.00%) | 424 (45.99%) |  |
| **Physical activity, N (%)** |  |  | < 0.001 |
| **Less active** | 5814 (35.46%) | 533 (57.81%) |  |
| **Active** | 4886 (29.80%) | 219 (23.75%) |  |
| **Missing** | 5698 (34.75%) | 170 (18.44%) |  |
| **BMI (kg/m^2^)** | 30.47 ± 7.06 | 29.40 ± 7.14 | < 0.001 |
| **BMI categorical (kg/m^2^)** |  |  | < 0.001 |
| **< 25** | 3352 (20.44%) | 243 (26.36%) |  |
| **≥ 25, < 30** | 5346 (32.60%) | 318 (34.49%) |  |
| **≥ 30** | 7700 (46.96%) | 361 (39.15%) |  |
| **Low family income, N (%)** |  |  | 0.612 |
| **Yes** | 13061 (79.65%) | 728 (78.96%) |  |
| **No** | 3337 (20.35%) | 194 (21.04%) |  |
| **Hypertension, N (%)** |  |  | < 0.001 |
| **Yes** | 6302 (38.43%) | 181 (19.63%) |  |
| **No** | 10096 (61.57%) | 741 (80.37%) |  |
| **Dyslipidemia, N (%)** |  |  | 0.058 |
| **Yes** | 2838 (17.31%) | 182 (19.74%) |  |
| **No** | 13560 (82.69%) | 740 (80.26%) |  |
| **Pre-existing CVD, N (%)** |  |  | < 0.001 |
| **No** | 20801 (87.11%) | 567 (53.74%) |  |
| **Yes** | 3078 (12.89%) | 488 (46.26%) |  |
| **Diabetes, N (%)** |  |  | < 0.001 |
| **No** | 15910 (66.63%) | 624 (59.15%) |  |
| **Yes** | 7969 (33.37%) | 431 (40.85%) |  |

Notes: BMI, body mass index; CVD, cardiovascular disease; PSM, propensity score matching

* For continuous variables: P-value was by survey-weighted linear regression; For categorical variables: P-value was by survey-weighted Chi-square test

**Table S7** Characteristics of 17,320 participants according to diabetes after PSM

|  | **Non-diabetes** | **Diabetes** | **P-value^*^** |
| --- | --- | --- | --- |
| **N** | 9178 | 8142 |  |
| **All-cause mortality, N (%)** |  |  | 0.007 |
| **No** | 7588 (82.68%) | 6855 (84.19%) |  |
| **Yes** | 1590 (17.32%) | 1287 (15.81%) |  |
| **Cardiovascular mortality, N (%)** |  |  | 0.085 |
| **No** | 8664 (94.40%) | 7734 (94.99%) |  |
| **Yes** | 514 (5.60%) | 408 (5.01%) |  |
| **Follow-up (years)** | 7.85 ± 3.92 | 5.90 ± 3.46 | < 0.001 |
| **Age (years)** | 59.98 ± 13.58 | 59.62 ± 12.85 | 0.069 |
| **Age categorical (years)** |  |  | 0.023 |
| **< 65** | 5568 (60.67%) | 5077 (62.36%) |  |
| **≥ 65** | 3610 (39.33%) | 3065 (37.64%) |  |
| **Sex, N (%)** |  |  | < 0.001 |
| **Man** | 2917 (31.78%) | 2111 (25.93%) |  |
| **Woman** | 6261 (68.22%) | 6031 (74.07%) |  |
| **Race/Ethnicity, N (%)** |  |  | < 0.001 |
| **Mexican American** | 1081 (11.78%) | 1342 (16.48%) |  |
| **Other Hispanic** | 736 (8.02%) | 884 (10.86%) |  |
| **Non-Hispanic White** | 4584 (49.95%) | 3007 (36.93%) |  |
| **Non-Hispanic Black** | 1969 (21.45%) | 1937 (23.79%) |  |
| **Other Race** | 808 (8.80%) | 972 (11.94%) |  |
| **Education, N (%)** |  |  | 0.001 |
| **Less than high school** | 2492 (27.15%) | 2413 (29.64%) |  |
| **High school diploma** | 2170 (23.64%) | 1865 (22.91%) |  |
| **More than high school** | 4516 (49.20%) | 3864 (47.46%) |  |
| **Marital status, N (%)** |  |  | 0.935 |
| **Married or living with partner** | 5635 (61.40%) | 4994 (61.34%) |  |
| **Single** | 3543 (38.60%) | 3148 (38.66%) | - |
| **Drinking status, N (%)** |  |  | < 0.001 |
| **Nondrinkers** | 5485 (59.76%) | 4650 (57.11%) |  |
| **Drinkers** | 2984 (32.51%) | 2894 (35.54%) |  |
| **Missing** | 709 (7.72%) | 598 (7.34%) |  |
| **Smoking status, N (%)** |  |  | 0.030 |
| **Non-smokers** | 4593 (50.04%) | 3940 (48.39%) |  |
| **Smokers** | 4585 (49.96%) | 4202 (51.61%) |  |
| **Physical activity, N (%)** |  |  | < 0.001 |
| **Less active** | 3942 (42.95%) | 2405 (29.54%) |  |
| **Active** | 3468 (37.79%) | 1637 (20.11%) |  |
| **Missing** | 1768 (19.26%) | 4100 (50.36%) |  |
| **BMI (kg/m^2^)** | 29.72 ± 6.68 | 31.21 ± 7.40 | < 0.001 |
| **BMI categorical (kg/m^2^)** |  |  | < 0.001 |
| **< 25** | 2077 (22.63%) | 1518 (18.64%) |  |
| **≥ 25, < 30** | 3091 (33.68%) | 2573 (31.60%) | - |
| **≥ 30** | 4010 (43.69%) | 4051 (49.75%) |  |
| **Low family income, N (%)** |  |  | 0.028 |
| **No** | 7365 (80.25%) | 6424 (78.90%) |  |
| **Yes** | 1813 (19.75%) | 1718 (21.10%) |  |
| **Hypertension, N (%)** |  |  | 0.002 |
| **No** | 3535 (38.52%) | 2948 (36.21%) |  |
| **Yes** | 5643 (61.48%) | 5194 (63.79%) |  |
| **Dyslipidemia, N (%)** |  |  | 0.002 |
| **No** | 1679 (18.29%) | 1341 (16.47%) |  |
| **Yes** | 7499 (81.71%) | 6801 (83.53%) |  |

Notes: BMI, body mass index; CVD, cardiovascular disease; PSM, propensity score matching

* For continuous variables: P-value was by survey-weighted linear regression; For categorical variables: P-value was by survey-weighted Chi-square test

**Table S8** Characteristics of 17,320 participants according to pre-existing CVD after PSM

|  | **Without**  **pre-existing CVD** | **Pre-existing CVD** | **P-value^*^** |
| --- | --- | --- | --- |
| **N** | 13869 | 3451 |  |
| **All-cause mortality, N (%)** |  |  | < 0.001 |
| **No** | 12177 (87.80%) | 2266 (65.66%) |  |
| **Yes** | 1692 (12.20%) | 1185 (34.34%) |  |
| **Cardiovascular mortality, N (%)** |  |  | < 0.001 |
| **No** | 13407 (96.67%) | 2991 (86.67%) |  |
| **Yes** | 462 (3.33%) | 460 (13.33%) |  |
| **Follow-up (years)** | 7.11 ± 3.82 | 6.25 ± 3.82 | < 0.001 |
| **Age (years)** | 58.07 ± 13.04 | 66.80 ± 11.67 | < 0.001 |
| **Age categorical (years)** |  |  |  |
| **< 65** | 9287 (66.96%) | 1358 (39.35%) | < 0.001 |
| **≥ 65** | 4582 (33.04%) | 2093 (60.65%) |  |
| **Sex, N (%)** |  |  | < 0.001 |
| **Man** | 3576 (25.78%) | 1452 (42.07%) |  |
| **Woman** | 10293 (74.22%) | 1999 (57.93%) |  |
| **Race/Ethnicity, N (%)** |  |  | < 0.001 |
| **Mexican American** | 2104 (15.17%) | 319 (9.24%) |  |
| **Other Hispanic** | 1377 (9.93%) | 243 (7.04%) |  |
| **Non-Hispanic White** | 5716 (41.21%) | 1875 (54.33%) |  |
| **Non-Hispanic Black** | 3121 (22.50%) | 785 (22.75%) |  |
| **Other Race** | 1551 (11.18%) | 229 (6.64%) |  |
| **Education, N (%)** |  |  | < 0.001 |
| **Less than high school** | 3807 (27.45%) | 1098 (31.82%) |  |
| **High school diploma** | 3143 (22.66%) | 892 (25.85%) |  |
| **More than high school** | 6919 (49.89%) | 1461 (42.34%) |  |
| **Marital status, N (%)** |  |  | < 0.001 |
| **Married or living with partner** | 8742 (63.03%) | 1887 (54.68%) |  |
| **Single** | 5127 (36.97%) | 1564 (45.32%) | - |
| **Drinking status, N (%)** |  |  | 0.372 |
| **Nondrinkers** | 8132 (58.63%) | 2003 (58.04%) |  |
| **Drinkers** | 4677 (33.72%) | 1201 (34.80%) |  |
| **Missing** | 1060 (7.64%) | 247 (7.16%) |  |
| **Smoking status, N (%)** |  |  | < 0.001 |
| **Non-smokers** | 6461 (46.59%) | 2072 (60.04%) |  |
| **Smokers** | 7408 (53.41%) | 1379 (39.96%) |  |
| **Physical activity, N (%)** |  |  | < 0.001 |
| **Less active** | 4785 (34.50%) | 1562 (45.26%) |  |
| **Active** | 4150 (29.92%) | 955 (27.67%) |  |
| **Missing** | 4934 (35.58%) | 934 (27.06%) |  |
| **BMI (kg/m^2^)** | 30.42 ± 7.00 | 30.42 ± 7.33 | 0.990 |
| **BMI categorized (kg/m^2^)** |  |  | 0.032 |
| **< 25** | 2823 (20.35%) | 772 (22.37%) |  |
| **≥ 25, < 30** | 4554 (32.84%) | 1110 (32.16%) | - |
| **≥ 30** | 6492 (46.81%) | 1569 (45.47%) |  |
| **Low family income, N (%)** |  |  | < 0.001 |
| **Yes** | 11136 (80.29%) | 2653 (76.88%) |  |
| **No** | 2733 (19.71%) | 798 (23.12%) |  |
| **Hypertension, N (%)** |  |  | < 0.001 |
| **No** | 5755 (41.50%) | 728 (21.10%) |  |
| **Yes** | 8114 (58.50%) | 2723 (78.90%) |  |
| **Dyslipidemia, N (%)** |  |  | 0.123 |
| **No** | 2449 (17.66%) | 571 (16.55%) |  |
| **Yes** | 11420 (82.34%) | 2880 (83.45%) |  |

Notes: BMI, body mass index; CVD, cardiovascular disease; PSM, propensity score matching

* For continuous variables: P-value was by survey-weighted linear regression; For categorical variables: P-value was by survey-weighted Chi-square test

**Table S9** Subgroup analysis on the effects of the diabetes and pre-existing CVD in the risk of all-cause mortality using nondiabetic participants without pre-existing CVD as reference

|  | **DM ^+^ CVD ^-^** | | **DM ^-^ CVD ^+^** | | **DM ^+^ CVD ^+^** | |
| --- | --- | --- | --- | --- | --- | --- |
|  | **Crude model** | **Model 3** | **Crude model** | **Crude model** | **Crude model** | **Model 3** |
| **Age (years)** |  |  |  |  |  |  |
| **< 65** | **1.93 (1.64, 2.26)** | **1.92 (1.63, 2.27)** | **3.92 (3.16, 4.86)** | **2.33 (1.86, 2.91)** | **7.08 (5.86, 8.56)** | **4.48 (3.66, 5.50)** |
| **≥ 65** | 1.08 (0.97, 1.21) | **1.25 (1.12, 1.40)** | **2.05 (1.84, 2.28)** | **1.85 (1.66, 2.06)** | **2.37 (2.13, 2.65)** | **2.53 (2.25, 2.83)** |
| **Sex, N (%)** |  |  |  |  |  |  |
| **Man** | **1.67 (1.45, 1.93)** | **1.30 (1.12, 1.50)** | **4.07 (3.57, 4.64)** | **1.52 (1.33, 1.74)** | **4.91 (4.27, 5.65)** | **2.13 (1.83, 2.47)** |
| **Woman** | **1.75 (1.56, 1.97)** | **1.47 (1.30, 1.66)** | **4.56 (3.98, 5.22)** | **1.98 (1.72, 2.27)** | **6.19 (5.45, 7.03)** | **2.92 (2.55, 3.35)** |
| **Race/Ethnicity, N (%)** |  |  |  |  |  |  |
| **Mexican American** | **2.40 (1.81, 3.19)** | **1.84 (1.37, 2.47)** | **4.04 (2.55, 6.41)** | **2.11 (1.31, 3.37)** | **9.05 (6.58, 12.46)** | **3.74 (2.66, 5.27)** |
| **Other Hispanic** | **2.17 (1.51, 3.11)** | **1.47 (1.01, 2.13)** | **4.86 (2.95, 8.00)** | **1.93 (1.15, 3.26)** | **7.04 (4.64, 10.67)** | **2.77 (1.76, 4.37)** |
| **Non-Hispanic White** | **1.75 (1.55, 1.98)** | **1.35 (1.19, 1.53)** | **4.08 (3.66, 4.56)** | **1.65 (1.47, 1.85)** | **5.17 (4.57, 5.85)** | **2.32 (2.04, 2.64)** |
| **Non-Hispanic Black** | **1.75 (1.45, 2.10)** | **1.38 (1.14, 1.67)** | **3.50 (2.79, 4.39)** | **1.85 (1.46, 2.34)** | **4.97 (4.07, 6.06)** | **2.71 (2.18, 3.35)** |
| **Other Race** | **1.84 (1.20, 2.83)** | 1.23 (0.79, 1.92) | **5.22 (3.02, 9.02)** | 1.71 (0.97, 3.02) | **9.66 (6.07, 15.36)** | **2.69 (1.64, 4.43)** |
| **Education, N (%)** |  |  |  |  |  |  |
| **Less than high school** | **1.51 (1.31, 1.75)** | **1.37 (1.18, 1.60)** | **3.42 (2.91, 4.02)** | **1.57 (1.33, 1.85)** | **4.86 (4.17, 5.66)** | **2.62 (2.23, 3.07)** |
| **High school diploma** | **1.53 (1.28, 1.83)** | **1.41 (1.18, 1.70)** | **4.70 (3.94, 5.60)** | **2.10 (1.75, 2.53)** | **4.55 (3.78, 5.48)** | **2.49 (2.05, 3.03)** |
| **More than high school** | **1.68 (1.45, 1.95)** | **1.34 (1.15, 1.56)** | **4.74 (4.07, 5.51)** | **1.63 (1.39, 1.91)** | **6.20 (5.29, 7.28)** | **2.50 (2.12, 2.97)** |
| **Marital status, N (%)** |  |  |  |  |  |  |
| **Married or living with partner** | **1.81 (1.60, 2.06)** | **1.38 (1.21, 1.58)** | **4.82 (4.21, 5.51)** | **1.63 (1.42, 1.87)** | **6.51 (5.68, 7.47)** | **2.40 (2.07, 2.78)** |
| **Single** | **1.47 (1.29, 1.67)** | **1.30 (1.14, 1.48)** | **3.70 (3.25, 4.22)** | **1.79 (1.56, 2.05)** | **4.31 (3.78, 4.92)** | **2.42 (2.10, 2.78)** |
| **Smoking status, N (%)** |  |  |  |  |  |  |
| **Non-smokers** | **1.64 (1.46, 1.84)** | **1.43 (1.27, 1.62)** | **3.57 (3.17, 4.02)** | **1.75 (1.55, 1.98)** | **4.49 (3.98, 5.07)** | **2.47 (2.17, 2.80)** |
| **Smokers** | **1.69 (1.47, 1.94)** | **1.31 (1.14, 1.52)** | **5.39 (4.62, 6.28)** | **1.66 (1.42, 1.95)** | **6.93 (5.95, 8.07)** | **2.67 (2.27, 3.13)** |
| **BMI (kg/m^2^)** |  |  |  |  |  |  |
| **< 25** | **1.65 (1.38, 1.97)** | **1.31 (1.09, 1.56)** | **4.55 (3.90, 5.32)** | **1.66 (1.41, 1.95)** | **6.03 (4.91, 7.39)** | **2.55 (2.07, 3.14)** |
| **≥ 25, < 30** | **2.01 (1.72, 2.35)** | **1.41 (1.20, 1.65)** | **4.95 (4.23, 5.78)** | **1.75 (1.48, 2.06)** | **7.39 (6.26, 8.73)** | **2.56 (2.15, 3.05)** |
| **≥ 30** | **1.74 (1.51, 2.02)** | **1.41 (1.22, 1.64)** | **3.90 (3.26, 4.66)** | **1.79 (1.49, 2.15)** | **5.71 (4.93, 6.63)** | **2.58 (2.21, 3.02)** |
| **Low family income, N (%)** |  |  |  |  |  |  |
| **No** | **1.65 (1.49, 1.83)** | **1.36 (1.23, 1.51)** | **4.79 (4.31, 5.32)** | **1.77 (1.58, 1.97)** | **5.81 (5.22, 6.47)** | **2.54 (2.27, 2.84)** |
| **Yes** | **1.62 (1.34, 1.95)** | **1.42 (1.17, 1.72)** | **3.14 (2.54, 3.87)** | **1.56 (1.26, 1.94)** | **4.63 (3.80, 5.65)** | **2.57 (2.07, 3.19)** |
| **Hypertension, N (%)** |  |  |  |  |  |  |
| **No** | **1.62 (1.35, 1.94)** | **1.29 (1.07, 1.55)** | **6.33 (5.22, 7.67)** | **1.77 (1.44, 2.16)** | **9.94 (8.01, 12.34)** | **2.92 (2.32, 3.66)** |
| **Yes** | **1.31 (1.18, 1.45)** | **1.40 (1.26, 1.56)** | **2.90 (2.60, 3.23)** | **1.72 (1.54, 1.92)** | **3.46 (3.11, 3.86)** | **2.48 (2.22, 2.78)** |
| **Dyslipidemia, N (%)** |  |  |  |  |  |  |
| **No** | **1.67 (1.37, 2.04)** | **1.30 (1.06, 1.59)** | **4.77 (3.91, 5.81)** | **1.63 (1.33, 2.00)** | **6.28 (5.05, 7.80)** | **2.46 (1.96, 3.08)** |
| **Yes** | **1.69 (1.53, 1.87)** | **1.40 (1.26, 1.56)** | **4.38 (3.94, 4.88)** | **1.78 (1.59, 1.98)** | **5.61 (5.05, 6.24)** | **2.58 (2.31, 2.89)** |

Notes:

BMI, body mass index; CVD, cardiovascular disease; PSM, propensity score matching

For continuous variables: P-value was by survey-weighted linear regression; For categorical variables: P-value was by survey-weighted Chi-square test

Bold indicates statistical significance

**Table S10** Subgroup analysis on the effects of the diabetes and pre-existing CVD in the risks of cardiovascular mortality before PSM using nondiabetic participants without pre-existing CVD as reference

|  | **DM ^+^ CVD ^-^** | | **DM ^-^ CVD ^+^** | | **DM ^+^ CVD ^+^** | |
| --- | --- | --- | --- | --- | --- | --- |
|  | **Crude model** | **Model 3** | **Crude model** | **Model 3** | **Crude model** | **Model 3** |
| **Age (years)** |  |  |  |  |  |  |
| **< 65** | **2.59 (1.84, 3.64)** | **2.18 (1.53, 3.11)** | **6.20 (4.07, 9.43)** | **3.37 (2.18, 5.21)** | **12.94 (9.04, 18.53)** | **6.48 (4.39, 9.57)** |
| **≥ 65** | 1.02 (0.83, 1.25) | 1.17 (0.95, 1.44) | **2.97 (2.49, 3.54)** | **2.64 (2.20, 3.16)** | **3.38 (2.82, 4.06)** | **3.56 (2.94, 4.30)** |
| **Sex, N (%)** |  |  |  |  |  |  |
| **Man** | **1.51 (1.13, 2.01)** | 1.13 (0.84, 1.52) | **6.95 (5.59, 8.65)** | **2.41 (1.92, 3.03)** | **8.19 (6.48, 10.36)** | **3.29 (2.56, 4.23)** |
| **Woman** | **1.97 (1.57, 2.47)** | **1.51 (1.20, 1.91)** | **6.27 (4.93, 7.99)** | **2.42 (1.89, 3.10)** | **9.03 (7.20, 11.33)** | **3.68 (2.89, 4.67)** |
| **Race/Ethnicity, N (%)** |  |  |  |  |  |  |
| **Mexican American** | **3.22 (1.82, 5.68)** | **2.49 (1.38, 4.48)** | **3.30 (1.14, 9.59)** | 1.55 (0.52, 4.58) | **14.48 (7.92, 26.47)** | **5.85 (3.05, 11.21)** |
| **Other Hispanic** | **3.26 (1.58, 6.73)** | **2.14 (1.01, 4.56)** | **13.45 (6.09, 29.69)** | **5.76 (2.47, 13.40)** | **16.11 (7.70, 33.73)** | **6.59 (2.91, 14.93)** |
| **Non-Hispanic White** | **1.69 (1.33, 2.16)** | 1.25 (0.97, 1.59) | **6.07 (5.01, 7.35)** | **2.17 (1.78, 2.65)** | **8.21 (6.67, 10.09)** | **3.27 (2.63, 4.06)** |
| **Non-Hispanic Black** | **1.88 (1.31, 2.69)** | 1.36 (0.94, 1.96) | **6.13 (4.22, 8.90)** | **2.94 (1.99, 4.34)** | **6.82 (4.76, 9.75)** | **3.15 (2.14, 4.63)** |
| **Other Race** | 1.32 (0.50, 3.51) | 0.79 (0.29, 2.13) | **7.50 (2.85, 19.74)** | 2.62 (0.94, 7.31) | **14.30 (6.21, 32.92)** | **3.85 (1.57, 9.45)** |
| **Education, N (%)** |  |  |  |  |  |  |
| **Less than high school** | **1.83 (1.38, 2.42)** | **1.56 (1.17, 2.08)** | **5.18 (3.88, 6.92)** | **2.27 (1.68, 3.06)** | **8.72 (6.69, 11.35)** | **4.36 (3.30, 5.76)** |
| **High school diploma** | 1.41 (0.99, 2.00) | 1.18 (0.83, 1.69) | **6.92 (5.14, 9.31)** | **2.78 (2.04, 3.79)** | **5.12 (3.63, 7.20)** | **2.30 (1.61, 3.28)** |
| **More than high school** | **1.59 (1.18, 2.15)** | 1.24 (0.91, 1.69) | **7.90 (6.12, 10.20)** | **2.29 (1.75, 2.99)** | **10.03 (7.67, 13.14)** | **3.61 (2.71, 4.80)** |
| **Marital status, N (%)** |  |  |  |  |  |  |
| **Married or living with partner** | **1.84 (1.42, 2.37)** | **1.32 (1.01, 1.72)** | **7.87 (6.23, 9.93)** | **2.38 (1.87, 3.04)** | **10.20 (8.03, 12.96)** | **3.31 (2.55, 4.29)** |
| **Single** | **1.55 (1.22, 1.98)** | **1.32 (1.03, 1.69)** | **5.51 (4.41, 6.89)** | **2.46 (1.95, 3.10)** | **6.57 (5.26, 8.20)** | **3.40 (2.69, 4.31)** |
| **Smoking status, N (%)** |  |  |  |  |  |  |
| **Non-smokers** | **1.74 (1.37, 2.21)** | **1.42 (1.11, 1.81)** | **5.48 (4.41, 6.80)** | **2.51 (2.00, 3.14)** | **6.99 (5.61, 8.71)** | **3.39 (2.68, 4.28)** |
| **Smokers** | **1.69 (1.30, 2.18)** | 1.27 (0.98, 1.65) | **8.83 (6.94, 11.22)** | **2.33 (1.81, 2.99)** | **10.77 (8.45, 13.73)** | **3.62 (2.81, 4.67)** |
| **BMI (kg/m^2^)** |  |  |  |  |  |  |
| **< 25** | 1.33 (0.89, 2.00) | 1.02 (0.68, 1.54) | **7.44 (5.64, 9.83)** | **2.44 (1.83, 3.26)** | **11.05 (7.85, 15.55)** | **4.39 (3.10, 6.22)** |
| **≥ 25, < 30** | **1.99 (1.47, 2.69)** | 1.34 (0.99, 1.82) | **7.41 (5.69, 9.65)** | **2.29 (1.73, 3.02)** | **11.00 (8.30, 14.56)** | **3.37 (2.51, 4.54)** |
| **≥ 30** | **1.88 (1.43, 2.47)** | **1.49 (1.13, 1.97)** | **5.92 (4.40, 7.97)** | **2.59 (1.91, 3.52)** | **7.84 (6.04, 10.17)** | **3.34 (2.54, 4.37)** |
| **Low family income, N (%)** |  |  |  |  |  |  |
| **No** | **1.74 (1.42, 2.12)** | **1.37 (1.12, 1.68)** | **7.89 (6.61, 9.42)** | **2.64 (2.20, 3.18)** | **8.94 (7.42, 10.76)** | **3.54 (2.91, 4.30)** |
| **Yes** | **1.57 (1.09, 2.28)** | 1.27 (0.87, 1.87) | **3.77 (2.55, 5.57)** | **1.69 (1.13, 2.53)** | **7.19 (5.11, 10.12)** | **3.38 (2.32, 4.91)** |
| **Hypertension, N (%)** |  |  |  |  |  |  |
| **No** | **2.10 (1.44, 3.05)** | **1.63 (1.11, 2.37)** | **10.03 (6.95, 14.47)** | **4.25 (3.55, 5.10)** | **20.81 (14.41, 30.06)** | **5.45 (3.68, 8.06)** |
| **Yes** | 1.21 (0.99, 1.48) | **1.26 (1.03, 1.54)** | **4.25 (3.55, 5.10)** | **2.41 (2.00, 2.90)** | **4.73 (3.94, 5.68)** | **3.19 (2.63, 3.86)** |
| **Dyslipidemia, N (%)** |  |  |  |  |  |  |
| **No** | 1.26 (0.81, 1.96) | 0.95 (0.61, 1.48) | **6.54 (4.60, 9.31)** | **1.88 (1.31, 2.70)** | **11.09 (7.80, 15.75)** | **3.75 (2.60, 5.41)** |
| **Yes** | **1.84 (1.52, 2.24)** | **1.46 (1.20, 1.78)** | **7.02 (5.86, 8.43)** | **2.65 (2.19, 3.20)** | **8.40 (6.99, 10.11)** | **3.54 (2.91, 4.31)** |

Notes:

BMI, body mass index; CVD, cardiovascular disease; PSM, propensity score matching

For continuous variables: P-value was by survey-weighted linear regression; For categorical variables: P-value was by survey-weighted Chi-square test
